# Bilirubin levels and kidney function decline: An analysis of clinical trial and real world data

**DOI:** 10.1371/journal.pone.0269970

**Published:** 2022-06-21

**Authors:** Yasunori Aoki, Claudia S. Cabrera, Mario Ouwens, Krister Bamberg, Jenny Nyström, Itamar Raz, Benjamin M. Scirica, Bengt Hamrén, Peter J. Greasley, Dinko Rekić

**Affiliations:** 1 Clinical Pharmacology and Safety Sciences, AstraZeneca, Gothenburg, Sweden; 2 Real World Science and Digital, BioPharmaceuticals Medical, AstraZeneca, Gothenburg, Sweden; 3 Biometrics Oncology, AstraZeneca, Gothenburg, Sweden; 4 Early Clinical Development, Research and Early Development, Cardiovascular, Renal, and Metabolism (CVRM) BioPharmaceuticals R&D, AstraZeneca, Gothenburg, Sweden; 5 The Sahlgrenska Academy at University of Gothenburg, Gothenburg, Sweden; 6 Hadassah University Hospital, Jerusalem, Israel; 7 Brigham and Women’s Hospital Heart & Vascular Center, Boston, Massachusetts, United States of America; 8 Harvard Medical School, Boston, Massachusetts, United States of America; Nazarbayev University School of Medicine, KAZAKHSTAN

## Abstract

**Objective:**

To evaluate if previously found associations between low serum bilirubin concentration and kidney function decline is independent of hemoglobin and other key confounders.

**Research design and methods:**

Clinical trial data from the SAVOR-TIMI 53 trial as well as the UK primary care electronic healthcare records, Clinical Practice Research Datalink (CPRD), were used to construct three cohorts of patients at risk of chronic kidney disease (CKD). The randomized clinical trial (RCT) cohort from the subset of SAVOR-TIMI 53 trial consisted of 10,555 type-2 diabetic patients with increased risk of cardiovascular disease. The two observational data cohorts from CPRD consisted of 71,104 newly diagnosed type-2 diabetes (CPRD-DM2) and 82,065 newly diagnosed hypertensive (CPRD-HT) patients without diabetes. Cohorts were stratified according to baseline circulating total bilirubin levels to determine association on the primary end point of a 30% reduction from baseline in estimated glomerular filtration rate (eGFR) and the secondary end point of albuminuria.

**Results:**

The confounder adjusted hazard ratios of the subpopulation with lower than median bilirubin levels compared to above median bilirubin levels for the primary end point were 1.18 (1.02–1.37), 1.12 (1.05–1.19) and 1.09 (1.01–1.17), for the secondary end point were 1.26 (1.06–1.52), 1.11 (1.01–1.21) and 1.18 (1.01–1.39) for SAVOR-TIMI 53, CPRD-DM2, CPRD-HT, respectively.

**Conclusion:**

Our findings are consistent across all cohorts and endpoints: lower serum bilirubin levels are associated with a greater kidney function decline independent of hemoglobin and other key confounders. This suggests that increased monitoring of kidney health in patients with lower bilirubin levels may be considered, especially for diabetic patients.

## 1. Introduction

Emerging treatment options for chronic kidney disease (CKD) require clinicians to make decisions on which treatments and when to start them. This is particularly critical given that the stage of disease at diagnosis and the kidney function decline can vary significantly between patients. Current approaches to identifying CKD patients early, especially those most likely to progress quicker are limited.

Bilirubin is a degradation product of hemoglobin that is excreted following conjugation by glucuronosyltransferase 1A1 (UGT1A1) into the bile duct and is used as a marker of liver function or hemolytic diseases. Recently, bilirubin has gained attention as an endocrine molecule rather than just a waste product of heme metabolism; besides well-known antioxidative effects, bilirubin has been shown to have antithrombotic, lipid lowering, immunomodulating, and insulin sensitizing properties [1, 2]. Observational clinical studies have shown that subjects with mildly elevated circulating bilirubin levels have a reduced risk for cardiovascular disease, type 2 diabetes, and respiratory disease [3–5].

Similarly, other observational studies have shown associations between naturally elevated circulating bilirubin levels and a slower CKD progression in patients with and without diabetes [1, 6–28]. This association has however been mainly demonstrated in Asian populations; hence, it is uncertain if the results can be expanded to non-Asian populations. To evaluate a potential association between circulating bilirubin levels on CKD progression and outcomes it is important to account for known factors impacting bilirubin levels as well as CKD. For example, bilirubin and hemoglobin levels are highly associated; low bilirubin may therefore be a consequence of anemia commonly seen in CKD. However, prior to this investigation, only Mashitani et al. accounted for hemoglobin when evaluating the association between circulating bilirubin levels and CKD, showing that the associations on CKD outcomes were statistically insignificant. Consequently, the question on the association of bilirubin versus hemoglobin levels on CKD outcomes and their applicability to non-Asian populations remains unanswered.

To evaluate the association of circulating bilirubin levels with development of CKD in subjects with varying risks (established diabetes with additional cardiovascular risk, newly diagnosed diabetes, and newly diagnosed hypertension), we compiled the largest data set to date, consisting of 163 724 racially diverse patients using a mix of real-world data from primary care electronic health records in the United Kingdom (UK) and data from a well-controlled clinical trial (SAVOR-TIMI 53) [29].

## 2. Methods

### 2.1. Data source

We conducted a retrospective analysis of a cohort from the study of Saxagliptin Assessment of Vascular Outcomes Recorded in Patients with Diabetes Mellitus (SAVOR)-Thrombolysis in Myocardial Infarction (TIMI) 53 [29] and two cohorts selected from the Clinical Practice Research Datalink (CPRD).

The SAVOR-TIMI 53 trial was a multicenter phase 4 clinical trial to evaluate the efficacy of saxagliptin on major adverse cardiovascular events (MACE) in patients with type 2 diabetes. The SAVOR-TIMI 53 trial included patients with established cardiovascular disease or multiple MACE risk factors. A subset of the dataset including both placebo and active arms was approved for this secondary analyses (excluding patients who did not sign the informed consent form for secondary use of data and the patients from countries where secondary usage of a dataset is not permitted). The secondary usage of this dataset was approved by AstraZeneca (study code: INT-20190919-235).

The CPRD database contains coded and anonymized electronic health records from primary care practices. CPRD offers a quality-assured source of longitudinal and representative real-time UK population health data for epidemiological and pharmaco-epidemiological research. The CPRD is representative of the general UK population in terms of age, sex and ethnicity [30–32] CPRD includes a UK-wide network of over 1,300 primary care practices and includes over 35 million patient records of which 11 million are currently registered active patients with at least 20 years of follow-up for 25% of the patients. (from https://www.cprd.com/ accessed on April 4th, 2019) CPRD requires the approval of the study protocol including statistical analyses plan prior to the release of the data hence all our analyses on CPRD is based on the prespecified approved analyses plan. Ethical approval and study protocol approval were granted by the CPRD scientific committee and the National Information Governance Board of Ethics and Confidentiality Committee (Approved by Independent Scientific Advisory Committee for MHRA database research ID number 19_144, protocol attached as a S1 File).

### 2.2. Patients

**Fig 1:**
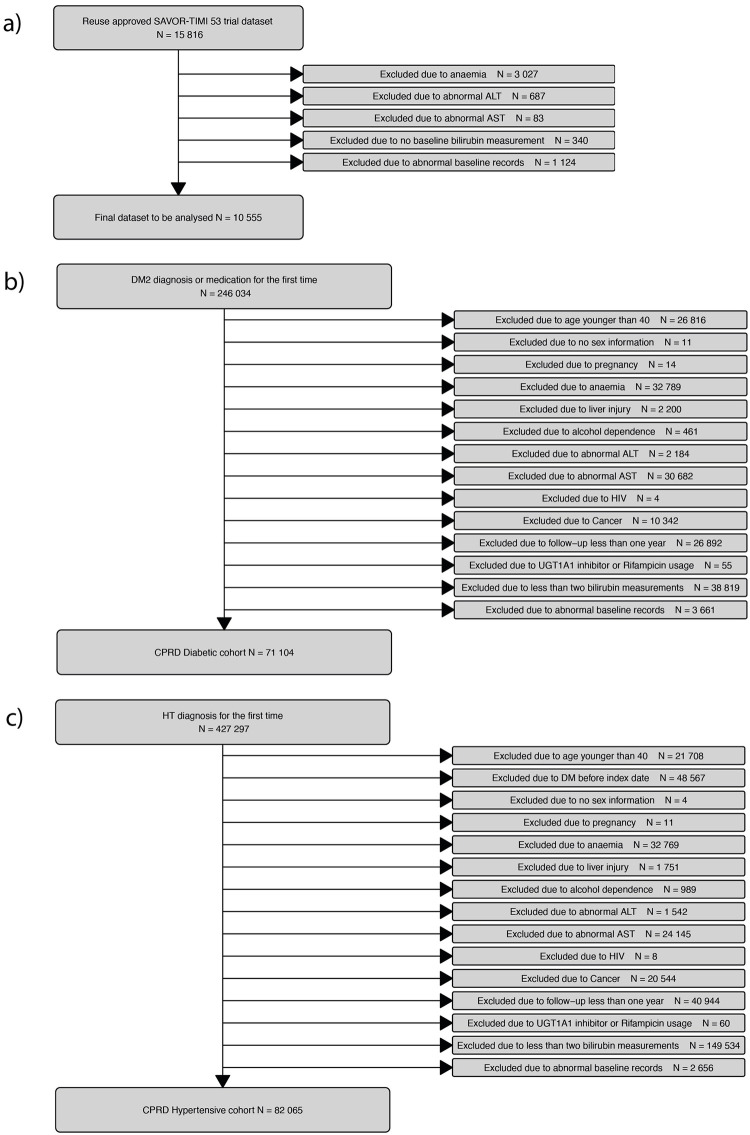
Flow diagram of patient disposition. The study cohorts are SAVOR (a subset of The Saxagliptin Assessment of Vascular Outcomes Recorded in Patients with Diabetes Mellitus-Thrombolysis in Myocardial Infarction 53) (Panels a), CPRD-DM2 (Type 2 diabetic cohort constructed from Clinical Practice Research Datalink) (Panels b), CPRD-DM2 (Hypertensive cohort constructed from Clinical Practice Research Datalink) (Panel c).

We used the dataset of randomized patients in the SAVOR-TIMI 53 trial with consent for secondary data analyses (n = 15,816). The baseline was defined as the values recorded from the visit immediately prior to randomization. In total 5,261 (33%) subjects were removed from the analysis dataset; the most common reason being anemia (n = 3,027, 19%) followed by liver abnormality (n = 770, 5%), lack of bilirubin measurement at baseline (n = 340, 2%) or other abnormalities at the baseline (See Fig 1 for the complete patient disposition diagram with detailed exclusion criteria.). Anemia is defined as hemoglobin less than 120g/L for female and less than 130g/L for men. Liver abnormality is defined as outside of the normal range of AST and ALT (AST: 8-48U/L, ALT: 7-55U/L). In this paper we refer this subset of dataset of the SAVOR-TIMI 53 trial as “SAVOR cohort”.

The CPRD dataset was used to constructed two cohorts at risk for decreasing kidney function: a cohort of patients with newly diagnosed type 2 diabetes (CPRD-DM2) and a cohort of non-diabetic patients with newly diagnosed primary hypertension (CPRD-HT) without diabetes. In CPRD-DM2, all patients with diagnoses dates of type 2 diabetes between January 2005 and January 2016 and with a clinical record at least one year prior and three years after the diagnoses date were included. The type 2 diabetes diagnosis was defined by the presence of a clinical record of Read code [33] subchapter C10, or two prescriptions of oral anti-diabetic medication or one glycated hemoglobin (HbA1c) lab value greater than 6.5%. The baseline values for the continuous covariates are defined as the average of all the measurements available during the acute disease period (within two years following diagnoses). The baseline value used for urine protein is defined as the greatest value observed using dipstick during the acute disease period. Out of 246,034 patients with type 2 diabetes diagnosis, 71,104 (29%) met the additional inclusion criteria for the CPRD-DM cohort. Lack of bilirubin measurements at baseline (n = 38,819, 16%), liver-related comorbidity (n = 35,527, 14%) and anemia (n = 32,789, 13%) were the most common reasons for not meeting the inclusion criteria. See Fig 1 for the complete patient disposition diagram and attached study protocol for the technical definitions of these exclusion criteria.

In CPRD-HT, all patients with a first clinical record of primary hypertension defined by Read code subchapter G2 between January 2005 and January 2016 were included. Of the available 427,297 patients with a date of first diagnoses of primary hypertension, 82,065 (19%) met the remaining inclusion criteria. The main reasons for not meeting the inclusion criteria was lack of sufficient bilirubin measurements at baseline (n = 149,534, 35%), prior diagnosis of diabetes to hypertension diagnosis (n = 48,567, 11%), insufficient follow-up (n = 40,944, 10%) and anemia (n = 32,769, 8%). See Fig 1 for the complete patient disposition diagram.

### 2.3. Measurers

#### 2.3.1. Exposure

Patients were divided into either high or low circulating total bilirubin groups using the population median levels at baseline. For the SAVOR cohort, the baseline measurements were defined as the lab measurements and clinical observations immediately before randomization. For CPRD the baseline serum bilirubin levels were determined as the average of all available measurements during the acute disease period (2 years after the rolling start of the study date, cf. S1 Fig). At least two bilirubin measurements were required to obtain the average. We refer to the group with greater than or equal to median circulating bilirubin levels (9μmol/L for SAVOR and 10μmol/L for CPRD-DM2 and CPRD-HT) as the ‘high bilirubin group’ and the group with less than median circulating total bilirubin levels as the ‘low bilirubin group’.

#### 2.3.2. Outcome (UACR and eGFR outcome definitions)

The primary endpoint was the first observation of an estimated glomerular filtration rate (eGFR) (calculated using the Chronic Kidney Disease Epidemiology Collaboration equation (CKD-EPI) [34]) decrease of greater than 30% from baseline. For the CPRD cohorts we required a sustained 30% reduction, defined as more than two consecutive 30% decreases from baseline. For the SAVOR cohort, we count the single occurrence of a 30% decrease as an end point (due to the lower frequency of observations for each patient). Subjects without baseline eGFR (CKD-EPI) were excluded.

The secondary end point was the first observation of urine albumin creatinine ratio (UACR) greater than 30mg/mmol. Due to the frequency of available UACR measurements, we used a single occurrence of such event as an end point. Patients without UACR records at baseline were included, whilst patients with a baseline UACR greater than 30mg/mmol were exclude.

### 2.4. Statistical analysis

Baseline variables were summarized for each cohort and high/low bilirubin group.

The standardized difference for each covariate with respect to the high/low bilirubin groups was calculated to identify potential imbalanced covariates between each group [35]. A directed acyclic graph (DAG) analysis of covariates was conducted to identify confounders and mediators among available baseline covariates [36, 37]. In DAG analyses, we first created nodes for all the covariates included in the analyses, then second joined the nodes with directed edge by the known or suspected causal effect (regardless of the effect size). Once the diagram is generated, each covariate is assessed either if it is a mediator or a confounder by applying the definition (if the covariate has causal effects to both bilirubin and endpoint then it is a confounder, if the bilirubin has causal effect to the covariate and the covariate has the causal effect to endpoint then it is a mediator).

A multivariable logistic regression model with confounder (and not mediator) as covariates was used to calculation the propensity scores. Then the propensity scores of high bilirubin are used to calculate stabilized inverse probability weights (IPW). More specifically for the individual in the high bilirubin group, the IPW is calculated by the probability of the patient in the high bilirubin group divided by the propensity score (the probability of patient in the high bilirubin group given the covariate calculated based on the logistic regression analysis). The IPW for the individual in the low bilirubin group can be calculated similarly.

The covariate balance between the low and high bilirubin groups after IPW was diagnosed by recalculating the standardized differences with IPW.

Multivariable Cox’s proportional hazard model with confounder (and not mediator) as covariate with stabilized inverse probability weighting scheme [35] was used. We included confounders identified by the DAG analysis in the final multivariable Cox model. Missing covariates were handled using the missing indicator method [38] so that the propensity score can be calculated.

To account for between-subject correlations we applied a case sampling bootstrap method to compute the mean and variance of the HR and computed the confidence interval. All the analyses are done for each cohort separately.

### 2.5. Sensitivity analyses

The analysis of the primary and secondary end points was conducted with and without use of IPW and adjustment for baseline risk factors. Sensitivity of the result to the definition of high/low bilirubin groups was evaluated by dividing subjects into 5 or 10 bilirubin quantiles and computed the HR considering the highest serum bilirubin level group as the reference. Prespecified subgroup analyses were performed to identify the interaction between the subgroup and the hazard ratios. The followings are predefined subgroups: younger or older than 75 years old, male or female, smoker or no-smoker, CKD stages, albuminuria stages, ACE inhibitor use, statin use, diuretic use, pottasium sparing diuretics use and race.

## 3. Results

### 3.1. Patient characteristics

The baseline characteristics of each cohort and high/low bilirubin group are described in Table 1. The SAVOR cohort had the highest HbA1c level whilst CPRD-HT had the lowest. Mean eGFR was comparable across all three cohorts; however, UACR was significantly higher in the SAVOR cohort. Both CPRD cohorts had a higher proportion of females than the SAVOR cohort. The CPRD cohorts had a slightly greater proportion of smokers than the SAVOR cohort.

**Table 1:** Characteristics of the patients at baseline*.

		SAVOR		CPRD DM2		CPRD HT	
Characteristic		Low Bilirubin < 9μmol/L	High Bilirubin ≥9μmol/L	Low Bilirubin < 10μmol/L	High Bilirubin ≥ 10μmol/L	Low Bilirubin < 10μmol/L	High Bilirubin ≥ 10μmol/L
**n**							
		4937	5618	35716	35388	40996	41069
**Serum Bilirubin (μmol/L)**							
	mean ±sd	6.07 ±1.00	11.65 ±3.67	7.45 ±1.48	14.05 ±3.98	7.49 ±1.45	13.97 ±3.95
**Age (years)**							
	mean ±sd	64.39 ±8.14	65.46 ±8.25	62.96 ±11.19	65.06 ±11.11	62.61 ±10.95	63.78 ±10.94
**>= 75years**	(%)	561 (11.4)	824 (14.7)	5919 (16.6)	7649 (21.6)	6215 (15.2)	7221 (17.6)
**Sex**							
**Female**	(%)	1955 (39.6)	1340 (23.9)	20535 (57.5)	12426 (35.1)	26547 (64.8)	16440 (40.0)
**Race**							
**White**	(%)	3862 (78.2)	4426 (78.8)	-	-	-	-
**Asian**		351 (7.1)	609 (10.8)	-	-	-	-
**Black or African American**		168 (3.4)	90 (1.6)	-	-	-	-
**Other**		556 (11.3)	493 (8.8)	-	-	-	-
**Body Mass Index (kg/m^2)**							
	mean ±sd	31.60 ±5.55	30.80 ±5.13	31.72 ±5.97	30.48 ±5.42	29.24 ±5.52	28.60 ±4.93
**>= 30**	(%)	2830 (57.3)	2870 (51.1)	18285 (51.2)	15375 (43.4)	11000 (26.8)	9216 (22.4)
**eGFR CKDEPI**							
	mean ±sd	75.74 ±19.90	75.92 ±18.29	79.38 ±17.69	77.59 ±16.97	77.82 ±16.43	76.83 ±15.72
**Stage 1 (>= 90ml/min/1.73m2)**	(%)	1423 (28.8)	1426 (25.4)	10893 (30.5)	8984 (25.4)	10351 (25.2)	8922 (21.7)
**Stage 2 (60-90ml/min/1.73m2)**		2381 (48.2)	3044 (54.2)	19452 (54.5)	20717 (58.5)	24368 (59.4)	25785 (62.8)
**Stage 3a (45-60ml/min/1.73m2)**		739 (15.0)	812 (14.5)	4058 (11.4)	4441 (12.5)	4952 (12.1)	5103 (12.4)
**Stage 3b (30-45ml/min/1.73m2)**		311 (6.3)	284 (5.1)	1142 (3.2)	1101 (3.1)	1080 (2.6)	970 (2.4)
**Stage 4 (15-30ml/min/1.73m2)**		77 (1.6)	47 (0.8)	133 (0.4)	108 (0.3)	96 (0.2)	76 (0.2)
**Stage 5 (<15ml/min/1.73m2)**		2 (0.0)	0 (0.0)	3 (0.0)	3 (0.0)	3 (0.0)	2 (0.0)
**Urine Albumin Creatinine ratio (mg/mmol)**							
	mean ±sd	17.62 ±65.92	13.07 ±54.53	4.69 ±65.08	3.16 ±12.93	6.65 ±53.93	4.02 ±17.73
**<3mg/mmol**	(%)	2962 (60.0)	3561 (63.4)	16044 (44.9)	16258 (45.9)	2715 (6.6)	2514 (6.1)
**3-30mg/mmol (Microalbuminuria)**		1350 (27.3)	1500 (26.7)	4172 (11.7)	3640 (10.3)	1036 (2.5)	833 (2.0)
**>30mg/mmol (Macroalbuminuria)**		494 (10.0)	419 (7.5)	418 (1.2)	268 (0.8)	110 (0.3)	53 (0.1)
**HbA1c (%)**							
	mean ±sd	8.00 ±1.35	7.84 ±1.30	7.10 ±1.07	6.93 ±1.04	5.72 ±0.36	5.66 ±0.39
**<6.5%**	(%)	349 (7.1)	482 (8.6)	10582 (29.6)	12991 (36.7)	6671 (16.3)	5959 (14.5)
**6.5 to <7.0%**		848 (17.2)	1106 (19.7)	10128 (28.4)	9426 (26.6)	17 (0.0)	12 (0.0)
**7.0 to <8.0%**		1628 (33.0)	1893 (33.7)	8718 (24.4)	7724 (21.8)	-	-
**8.0 to <9.0%**		979 (19.8)	1043 (18.6)	3453 (9.7)	3017 (8.5)	-	-
**>=9.0%**		1117 (22.6)	1074 (19.1)	2477 (6.9)	1846 (5.2)	-	-
**Hemoglobin (g/L)**							
	mean ±sd	140.05 ±10.69	144.81 ±10.74	141.81 ±10.25	146.66 ±10.52	140.30 ±10.07	145.44 ±10.63
**Smoking**							
**Smoker**	(%)	839 (17.0)	633 (11.3)	8891 (24.9)	4441 (12.5)	9830 (24.0)	5258 (12.8)
**AST (U/L)**							
	mean ±sd	20.85 ±6.16	22.24 ±6.59	24.59 ±7.64	25.75 ±7.62	23.20 ±7.68	24.77 ±7.81
**ALT (U/L)**							
	mean ±sd	24.00 ±9.35	25.71 ±9.83	22.53 ±6.07	24.54 ±6.40	23.29 ±5.68	25.01 ±5.80

* Plus-minus values are means ± standard deviations. High bilirubin denotes the patient subpopulation that has above or equal cohort median of the baseline serum bilirubin concentration (9μmol/L for SAVOR, 10μmol/L for CPRD-DM2 and HT), Low bilirubin denotes the patient subpopulation that has below cohort median of the baseline serum bilirubin concentration, eGFR CKDEPI estimated glomerular filtration rate using the formula by Chronic Kidney Disease Epidemiology Collaboration, HbA1c Glycosylated hemoglobin A1c, AST Aspartate aminotransferase, ALT Alanine aminotransferase.

^†^ Race was self-reported. ‘Other’ race in this table includes native Americans, pacific islanders, other race, and multiple race. Race information was not readily available in CPRD.

^‡^ Patients without baseline eGFR CKDEPI measurements are removed from the analyses of the primary endpoint of the study -estimated glomerular filtration rate (estimated by the CKD EPI formula) of more than 30% from the baseline-. Patients with evidence of albuminuria (urine albumin creatinine ratio greater or equal to 30g/mol) are removed from the analyses of the secondary endpoint of the study -first observation of the albuminuria (defined by the urine albumin creatinine ratio greater or equal to 30mg/mmol)-.

**Fig 2:**
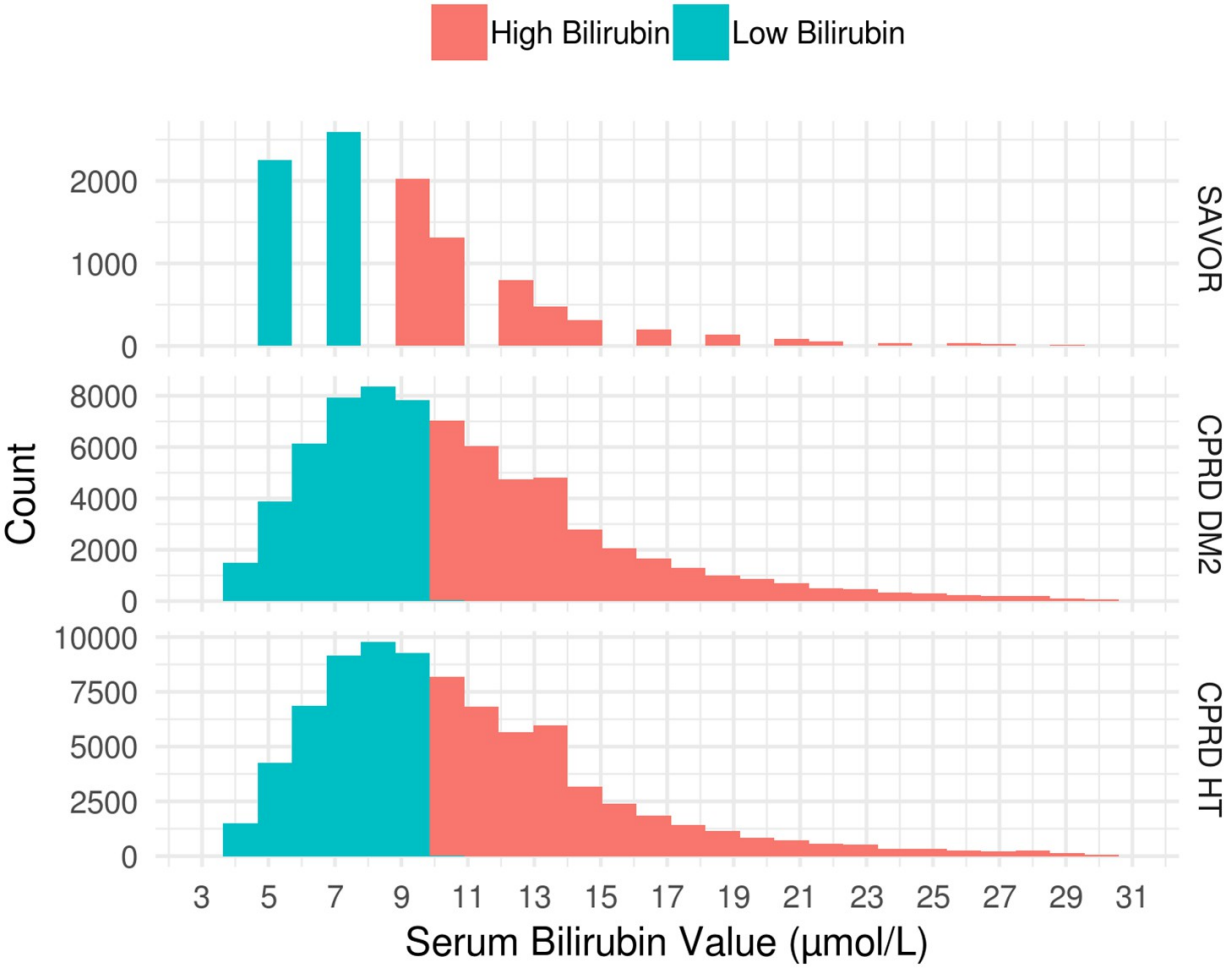
Distribution of baseline serum bilirubin according to study cohorts. Low bilirubin subpopulation is defined as the subpopulation with less than median baseline serum bilirubin concentration. High bilirubin subpopulation is defined by the patients with greater or equal to median serum bilirubin concentration at baseline. Population median of baseline serum bilirubin concentration was 9μmol/L for SAVOR and 10μmol/L for CPRD-DM2 and CPRD-HT.

**Fig 3:**
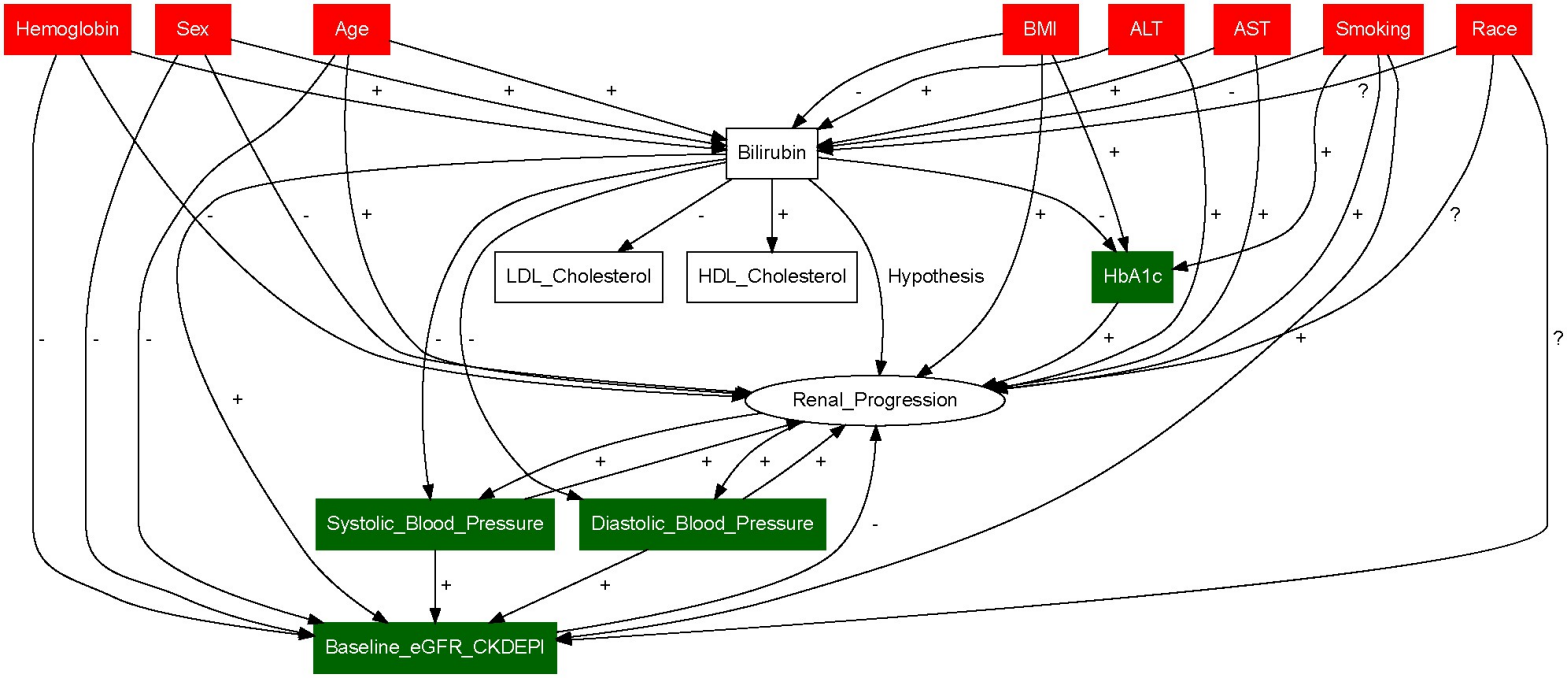
Directed Acyclic Graph (DAG) for the effect of serum bilirubin on renal progression. Arrows denote the known or suspected causal effect (regardless of the effect size). Red nodes are the covariates that are identified as confounders, and green nodes are the covariates that are identified to be mediators. As the total effects of the bilirubin are our interest, we adjust only for confounders and not for mediators. +—and? indicate the positive, negative and unspecific known or suspected causal association.

Distributions of Baseline serum bilirubin levels were similarly distributed across all cohorts (Fig 2). Factors identified as confounders through directed acyclic graph analyses were adjusted for, as the focus of this investigation was on the total association of bilirubin level on the risk of CKD (Fig 3). The identified confounders were hemoglobin, sex, age, body mass index (BMI), alanine transaminase (ALT), aspartate transaminase (AST), smoking and race, and they are used for the calculation of IPW. HbA1c, blood pressure and baseline eGFR were identified as mediators.

**Fig 4:**
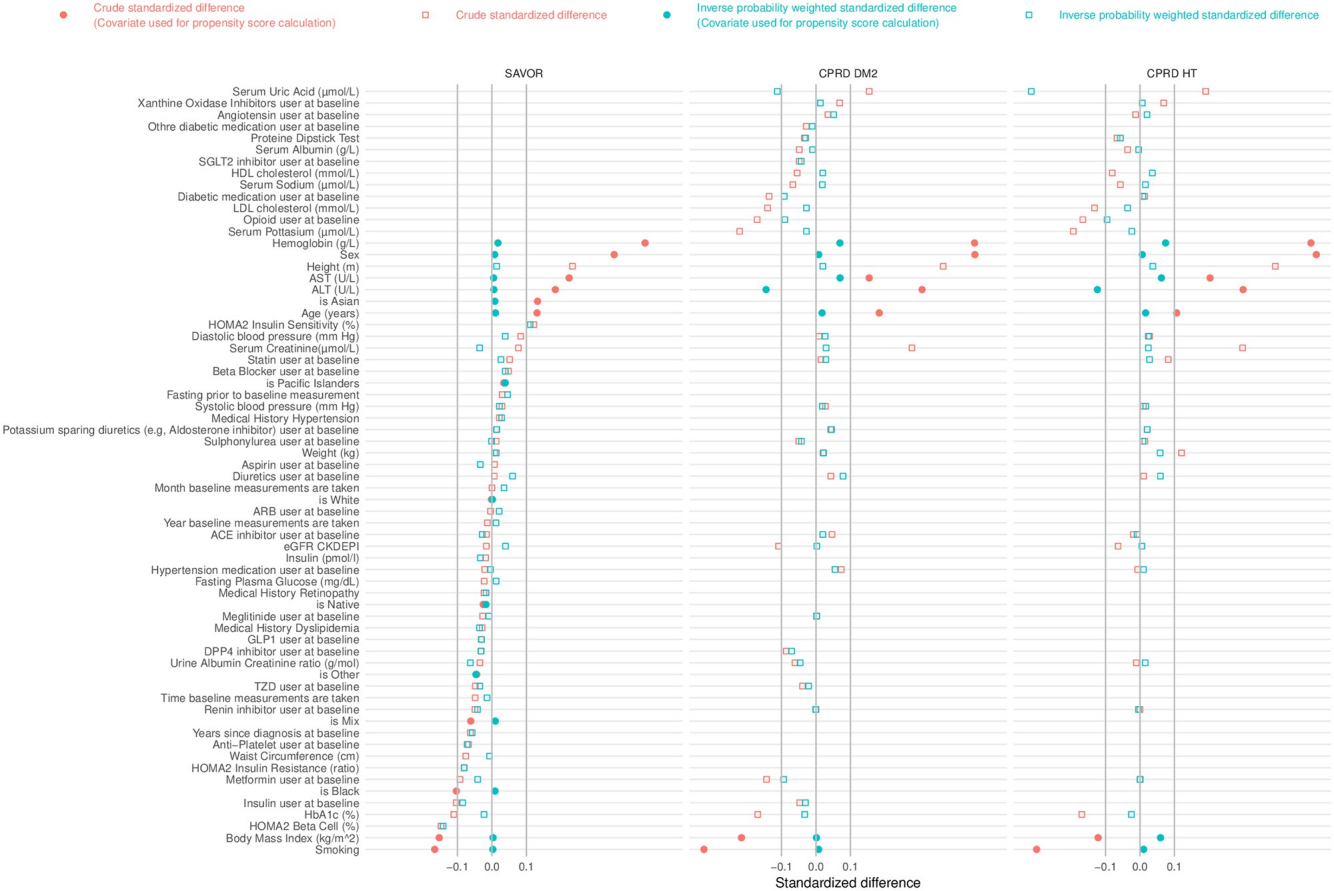
Standardized difference plot for each cohort with and without inverse probability weighting. Red dots and squares are the crude standardized difference of the patient subpopulation with high bilirubin (patients with greater than or equal to median baseline serum bilirubin value) against low bilirubin. Positive standardized difference indicates positive correlation between high bilirubin and the baseline characteristic listed in y-axis. We consider absolute standardized difference over 10% to be significantly different baseline characteristic between the high bilirubin group and low bilirubin group. Green dots and squares are the standardized difference after inverse probability weighting. Filled squares indicates these covariates (Sex, ALT, AST, Race, Age, Body Mass Index, Smoking) are used to calculate the propensity score for the inverse probability weighting. After the inverse probability weighting the difference of baseline characteristics between high bilirubin and low bilirubin subpopulations become not significant except HOMA2 Beta Cell, HOMA2 Insulin Sensitivity (for SAVOR), Serum Uric Acid and ALT (for CPRD cohorts). We note that the proportion of missing baseline records for HOMA2 Beta Cell, HOMA2 Insulin Sensitivity (for SAVOR), Serum Uric Acid and ALT (for CPRD-DM2) are 56%, 56%, 93%, 85%, respectively. Protein Dipstick Test results are converted in to the numerical values in the order of null, trace, +, ++ as 0,1,2,3 respectively. Sex is coded 0 as female and 1 as male. isAsian, isPacific Islander, isWhite, is Native, is Other, is Mix, is Black are the binary coding of the racial category of the patient, for example if the reported race of the patient is an Asian then 1 as isAsian variable and 0 for isPacific Islander, isWhite, isNative, isOther, isMix, and isBlack. Smoking is coded as a binary variable where 1 indicates the current smoker and 0 otherwise. Time baseline measurements are taken was recorded in 24 hr scale with minutes converted into fraction of an hour. Other diabetic medication is the medication included in the British National Formulary (BNF) code chapter ‘Other Antidiabetic Drugs’.

Fig 4 shows the standardized difference for investigated factors between subjects in high and the low bilirubin groups. Absolute standardized difference above 10% is an indication of a meaningful difference between groups [35]. Hemoglobin, male gender, height, AST, ALT, age, and Asian race had larger than 10% standardized difference in all cohorts. Insulin therapy, HbA1c, BMI, smoking, and black race had less than -10% of the standardized difference in all cohorts. After inverse probability weighting, all covariates except ALT and serum uric acid in the CPRD cohorts are within plus or minus 10% of standardized differences. However, ~80% of baseline ALT and ~95% of serum uric acid measurements are missing in CPRD resulting in this imbalance.

Fasting prior to baseline measurement is coded as a binary variable where if yes then coded as 1 and otherwise (no or unknown) 0.

### 3.2. Main outcome eGFR and UACR

The event rate for the primary end point of an eGFR decrease >30% was different in the three cohorts. As expected, subjects with established diabetes and additional CV risk factors in the SAVOR cohorts had an event rate of 42.8 per 1000 patient years (kPY), almost 5 times the event rate observed in the CPRD-DM2 cohort (9.2 per kPY) and 8 times the event rate observed in CPRD-HT cohorts (5.1 per kPY). These event rates differed even more between the cohorts for the secondary end point of albuminuria (SAVOR had 6- and 26-times higher event rate compared to CPRD DM and CPRD-HT cohorts respectively).

**Fig 5:**
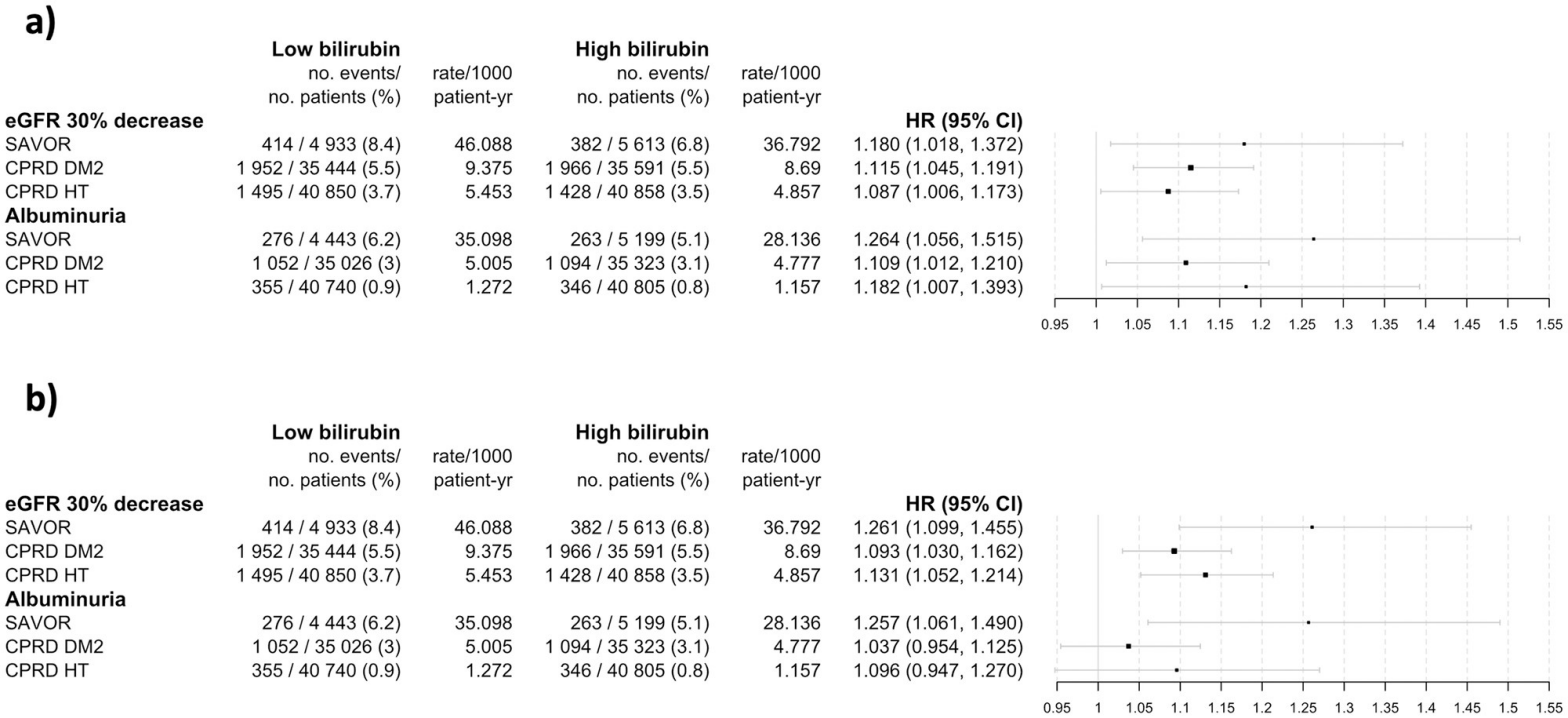
Primary and secondary endpoints, according to study cohorts. Hazard Ratio calculated with confounder adjustment and inverse probability weighting (panel a). Hazard Ratio calculated without any adjustment nor weighting (panel b). Shown is the primary endpoint of the study -estimated glomerular filtration rate (estimated by the CKD EPI formula) of more than 30% from the baseline- and the secondary endpoint of the study -first observation of the albuminuria (defined by the urine albumin creatinine ratio greater or equal to 30mg/mmol)- according to the study cohorts. High bilirubin denotes the patient subpopulation that has above or equal cohort median of the baseline serum bilirubin concentration (9μmol/L for SAVOR, 10μnil/L for CPRD-DM2 and HT), Low bilirubin denotes the patient subpopulation that has below cohort median of the baseline serum bilirubin concentration, HR hazard ratios in panel a, inverse probability weighted and adjusted for confounding covariates (age, sex, race if available, baseline body mass index, baseline hemoglobin, baseline alanine transaminase, baseline aspartate transaminase, smoking), HR hazard ratios in panel b, no inverse probability weighted and no adjustment, CI confidence interval calculated by fitting a normal distribution to the 1000 bootstrap sample of the HR.

**Fig 6:**
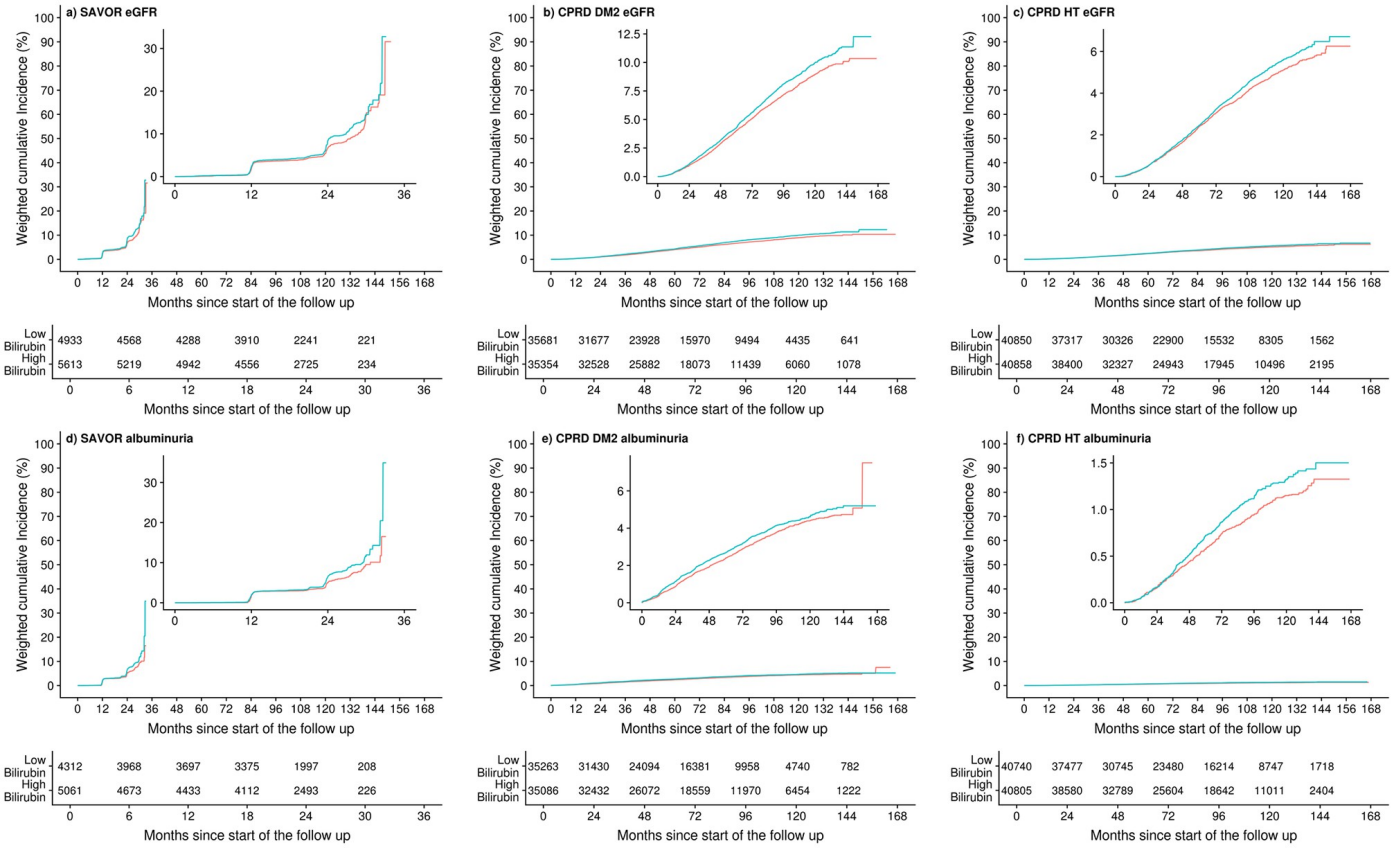
Weighted cumulative incidence plots of the primary and secondary end points. The primary endpoint of the study was estimated glomerular filtration rate (estimated by the CKD EPI formula) of more than 30% from the baseline (Panels a, b, c). The secondary endpoint of the study was the first observation of the albumiunria (defined by the urine albumin creatinine ratio greater or equal to 30mg/mmol) (Panels d, e, f). The study cohorts are SAVOR (Panels a, d), CPRD-DM2 (Panels b, e), CPRD- (Panels c, f). High bilirubin plotted in red are the patient subpopulation that has above or equal cohort median of the baseline serum bilirubin concentration (9μmol/L for SAVOR, 10μnil/L for CPRD-DM2 and HT), Low bilirubin plotted in blue are the patient subpopulation that has below cohort median of the baseline serum bilirubin concentration, The cumulative incidences were estimated with the use of the Kaplan–Meier method where the events are weighted by the inverse probability weighting scheme with the propensity score calculated based on the confounding covariates (age, sex, race if available, baseline body mass index, baseline hemoglobin, baseline alanine transaminase, baseline aspartate transaminase, smoking).

The results of the primary analyses are summarized in Fig 5a. The multivariable adjusted inverse probability-weighted hazard ratios (95% confidence interval) for the primary end point for SAVOR, CPRD-DM2, CPRD-HT were 1.18 (1.02–1.37), 1.12 (1.05–1.19), and 1.09 (1.01–1.17), respectively. The hazard ratio for the secondary end point for SAVOR, CPRD-DM2, CPRD-HT were 1.26 (1.06–1.52), 1.11 (1.01–1.21), and 1.18 (1.01–1.39), respectively. Weighted cumulative incidence of the primary and the secondary end points are shown in Fig 6 indicating consistently fewer weighted cumulated events in the high bilirubin group compared to the low bilirubin group.

### 3.3. Sensitivity analysis

The hazard ratios estimated from the multivariable cox proportional hazard models were almost identical between the analyses with or without the inverse probability weighting (S2 Fig). Without any confounder adjustment, all HRs were greater than one, and for all primary end points, they were statistically significant (Fig 5b).

**Fig 7:**
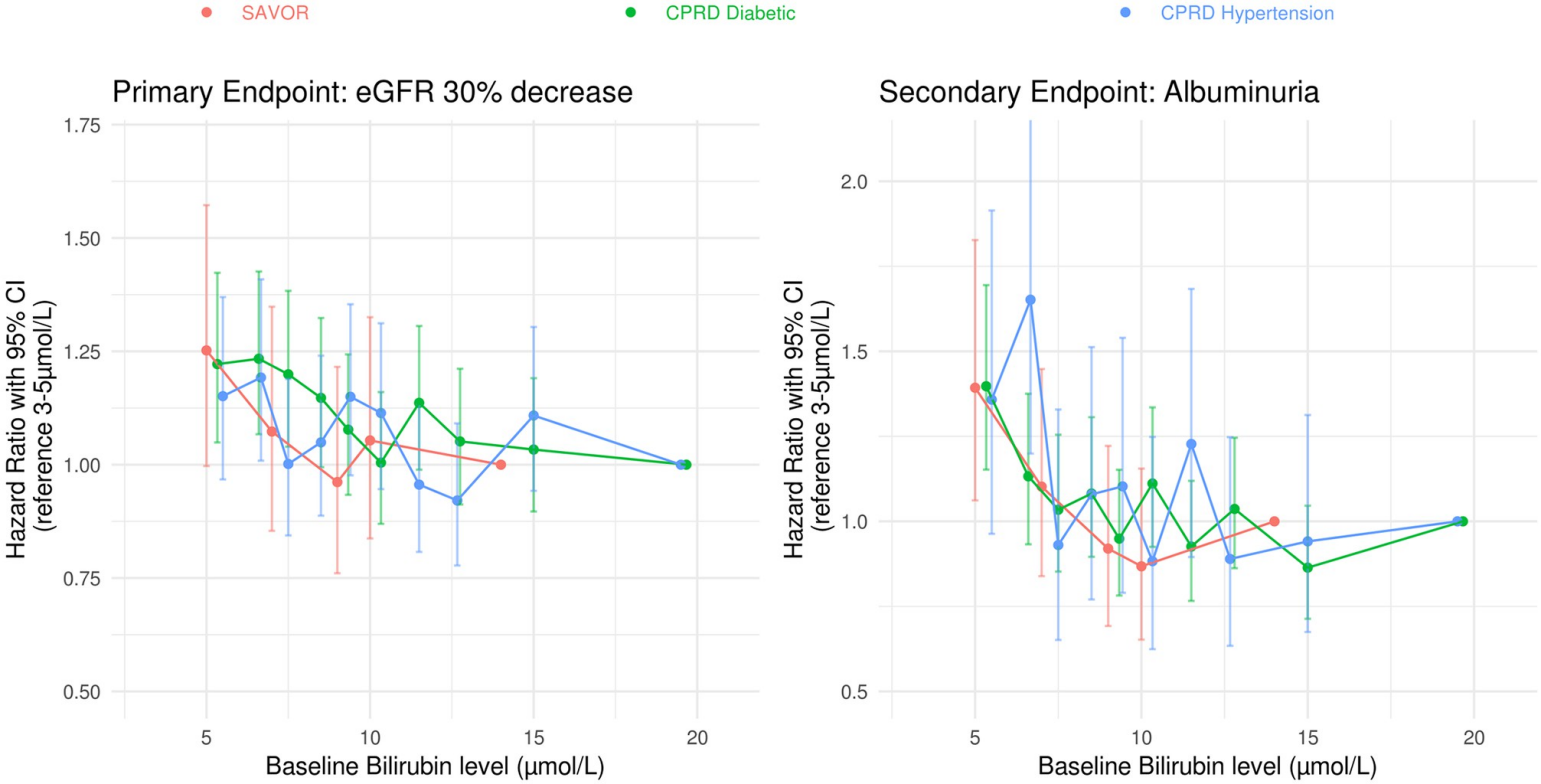
Relationship between the hazard ratio and serum bilirubin concentration. SAVOR cohort was subdivided into five quantiles by the baseline serum bilirubin level and the hazard ratios were calculated with the highest quantile as the reference. CPRD cohorts were subdivided into ten quantiles by the baseline serum bilirubin level and the hazard ratios were calculated with the highest quantile as the reference. Multivariable Cox proportional hazard model was used without inverse probability weighting to calculate the Hazard Ratios. The 95% confidence intervals for the outcomes were calculated based on robust variances.

In Fig 7, relationship between the hazard ratio and serum bilirubin concentration is plotted. The HRs were calculated relative to the highest serum bilirubin level group when the cohorts were divided into 5 or 10 quantiles. As can be seen in Fig 7, regardless of how the cohort is divided by the bilirubin level, a consistent relationship between lower bilirubin concentration and higher risk for the primary and the secondary end point.

The pre-specified subgroup analyses identified only age-group in the CPRD cohorts as statistically significant (S3a–S3f Fig). HR ratio for the primary and the secondary end points were lower in subjects below the age of 75 years in the CPRD cohorts and similar to those in the SAVOR cohort. HR ratios (95% confidence interval) for the primary end point in subjects below 75 years in SAVOR, CPRD-DM2, CPRD-HT were 1.18 (1.00–1.36), 1.19 (1.10–1.28), and 1.14 (1.04–1.24), respectively. The HR for the secondary end point for SAVOR, CPRD-DM2, CPRD-HT were 1.30 (1.08–1.56), 1.15 (1.04–1.27), and 1.33 (1.11–1.59), respectively.

## 4. Conclusion

To the best of our knowledge, the presented study is the most extensive retrospective study to date on the association between serum bilirubin and CKD. By using three distinct cohorts with different CKD risk, we have shown that low bilirubin level is an independent risk factor for kidney function decline in both diabetic and hypertensive patients.

This study was conducted using both registry and RCT data. The RCT data from the SAVOR cohort contributes a robust well-controlled cohort with standardized data collection and quality control. For example, fasting and time of day are known factors [39, 40] that influence bilirubin levels that previous investigations were unable to adjust for or standardize. However, in SAVOR, 97.4% of the baseline measurements were collected after fasting, and 95.3% of the samples were collected in the morning thus standardizing for the diurnal factors influencing bilirubin levels. In contrast, real-world data (RWD) offers a long-term view over a broad population in less controlled manner.

Similar to other studies we found that lower bilirubin levels were associated with an increased risk for kidney function decline and by extension, CKD. Importantly, this finding was consistent in both a well-controlled RCT setting as well as in RWD. Most of the prior analyses evaluating the levels of bilirubin on CKD risk were conducted in Asian populations with only one study by Riphagen et al. [41] that included a considerable number of Caucasians. However, the study by Riphagen et al. [41] focused on the risk for progression in already established CKD in contrast to our study where we demonstrate the association of lower bilirubin levels for the wide range of CKD risk populations. Our study, therefore provides an essential extension of the previously reported findings.

The study of Mashitani et al^20^ was based on 957 subjects with microalbuminuria and demonstrated a reduced risk for CKD progression among subjects with elevated bilirubin levels [25]. However, after adjusting for baseline hemoglobin levels, results were no longer statistical significant. Since bilirubin is a catabolic product of heme breakdown, it might be inferred that lower bilirubin levels reflect lower hemoglobin levels associated with CKD progression. Hence, from their study it is not possible to conclude if serum bilirubin level is independently associated with CKD risk, or if it was merely a confounding factor of hemoglobin. Mashitani and colleagues’ study was replicated in various other East Asian populations; none of these studies provided the answer to this question. Our study, therefore, is designed to provide evidence to show whether the association between the CKD risk and serum bilirubin is independent from the hemoglobin level.

The size of the estimated relative risk was largest in the SAVOR cohort that included patients at highest risk for CKD (18% higher risk) and smallest in CPRD-HT cohort with the lowest risk (8.7% higher risk). However, the difference between the estimated HR ratios was profoundly smaller in the prespecified subgroup analysis of subjects less than 75 years of age, (18% versus 14%). The concentration versus HR analysis of bilirubin (Fig 7) shows that compared to the highest quantile subjects, the patients in lowest quantile have ~30% higher risk for both the primary and the secondary end points.

Based on these results, we conclude that an association between low serum bilirubin levels and an elevated risk of CKD can be extended beyond Asian populations. All these results are statistically significant while being adjusted for the key confounders such as smoking and hemoglobin levels. We therefore conclude that low bilirubin levels are an independent CKD risk factor and not a consequence of lower hemoglobin levels associated with CKD.

Since our study is an observational study there are several limitations. Firstly, the results presented in this study were adjusted for known and measured confounders. However, there is a possibility that the associations presented here could be due to other unavailable factors such as participation in regular physical exercise. Secondly, CPRD is a real-word data set [31] with a significant number of missing variables (cf. S1 Table). We did not assume the missing variables to be missing at random and have therefore applied the missing indicator method in our analysis. We also removed all subjects without baseline bilirubin measurements which may have caused bias in the population. In addition, we have assumed that a no test for UACR at baseline as an indication of physicians overall judgement of normal kidney function. Hence for the CPRD cohorts, if the UACR measurements are not available at baseline we assumed the event (UACR >30mg/mmol) has yet to have occurred. In the CPRD cohorts, a significant number of patients did not have UACR measurements at baseline (CPRD-DM2, about 43%, CPRD-HT about 91%). On the other hand, for both CPRD-DM2 and CPRD-HT only about 2% of the patients with UACR measurements at baseline had UACR >30mg/mmol hence we believe the impact of above assumption is negligible. The limitations related to RWD mentioned here are to some extent mitigated by conducting the same analyses using RCT data where more precise measurements were performed on all subjects. Thirdly, in all of our datasets, genetic information that may explain individual variability of serum bilirubin level was not available; however if the genetic information was available, using the UGT 1A1 polymorphism data and conducting Mendelian randomization instead of dividing and comparing patients by baseline serum bilirubin levels would have been preferred. Despite these limitations, the results are consistent across all the cohorts with a wide range of CKD risks. Furthermore, the results were robust against the sensitivity analyses; hence, we believe this finding is generalizable to a broad patient population in the real world.

Physicians treating diabetes and hypertensive patients should consider that bilirubin measurements may be useful for early identification of patients at risk for kidney function decline and development of CKD. For example, patients with bilirubin <7 μmol/L may benefit from more frequent kidney function monitoring in order to detect kidney function decline earlier. Similarly, bilirubin may have utility in clinical trial patient enrichment in order to increase chances of detecting a treatment effect [42]. Finally, as recently indicated by Vitek et al., [2] and Hull and Agarwal [43], induction of a mild hyperbilirubinemia may present a therapeutic opportunity for several indications including CKD. Our studies have further supported that the elevation of bilirubin may be a therapeutic mechanism when seeking new treatments for CKD.

In conclusion, based on our retrospective analysis of the largest longitudinal cohort to date using RWD and RCT data, we suggest that the causality of low serum bilirubin levels to poor CKD outcomes should be investigated in future interventional studies.

## Supporting information

S1 File (DOCX)

S1 Table Number of patients with missing covariate records at baseline. Number of patients with missing covariate records at baseline. † Record not available for CPRD ‡ Record not available for SAVOR.
(DOCX)

S1 Fig Time related variable including the index date and the follow-up. (TIF)

S2 Fig Primary and secondary endpoints, according to study cohorts, hazard ratio calculated without inverse probability weighting. Shown is the primary endpoint of the study -estimated glomerular filtration rate (estimated by the CKD EPI formula) of more than 30% from the baseline- and the secondary endpoint of the study -first observation of the albuminuria (defined by the urine albumin creatinine ratio greater or equal to 30mg/mmol)- according to the study cohorts: SAVOR, a subset of The Saxagliptin Assessment of Vascular Outcomes Recorded in Patients with Diabetes Mellitus-Thrombolysis in Myocardial Infarction 53 trial, CPRD-DM2 (Type 2 diabetic cohort constructed from Clinical Practice Research Datalink), CPRD-HT (Hypertensive cohort constructed from Clinical Practice Research Datalink). High bilirubin denotes the patient subpopulation that has above or equal cohort median of the baseline serum bilirubin concentration (9μmol/L for SAVOR, 10μnil/L for CPRD-DM2 and HT), Low bilirubin denotes the patient subpopulation that has below cohort median of the baseline serum bilirubin concentration, HR hazard ratio adjusted for confounding covariates (age, sex, race if available, baseline body mass index, baseline hemoglobin, baseline alanine transaminase, baseline aspartate transaminase, smoking), CI confidence interval calculated by fitting a normal distribution to the 1000 bootstrap sample of the HR.
(TIF)

S3 Fig a. Prespecified subgroup analyses of Primary endpoint of SAVOR cohort, Hazard Ratio calculated with inverse probability weighting without multivariable adjustment. b. Prespecified subgroup analyses of Secondary endpoint of SAVOR cohort, Hazard Ratio calculated with inverse probability weighting without multivariable adjustment. c. Prespecified subgroup analyses of Primary endpoint of CPRD-DM2 cohort, Hazard Ratio calculated with inverse probability weighting without multivariable adjustment. d. Prespecified subgroup analyses of Secondary endpoint of CPRD-DM2 cohort, Hazard Ratio calculated with inverse probability weighting without multivariable adjustment. e. Prespecified subgroup analyses of Primary endpoint of CPRD-HT cohort, Hazard Ratio calculated with inverse probability weighting without multivariable adjustment. f. Prespecified subgroup analyses of Secondary endpoint of CPRD-HT cohort, Hazard Ratio calculated with inverse probability weighting without multivariable adjustment.
(TIF)

## References

[1] Tsai MT, Tarng DC. Beyond a measure of liver function—bilirubin acts as a potential cardiovascular protector in chronic kidney disease patients. Int J Mol Sci. 2019;20(1):1–19. 10.3390/ijms20010117 PMC6337523 30597982

[2] Vitek L, Bellarosa C, Tiribelli C. Induction of Mild Hyperbilirubinemia: Hype or Real Therapeutic Opportunity? Clin Pharmacol Ther. 2019;106(3). doi: 10.1002/cpt.1341 30588615

[3] Horsfall LJ, Rait G, Walters K, Swallow DM, Pereira SP, Nazareth I, et al. Serum Bilirubin and Risk of Respiratory Disease and Death [Internet]. http://www.jama. 10.1001/jama.2011.124 21325185

[4] Abbasi A, Deetman PE, Corpeleijn E, Gansevoort RT, Gans ROB, Hillege HL, et al. Bilirubin as a potential causal factor in type 2 diabetes risk: A mendelian randomization study. Diabetes. 2015. doi: 10.2337/db14-0228 25368098 PMC4346199

[5] Lin JP, O’Donnell CJ, Schwaiger JP, Cupples LA, Lingenhel A, Hunt SC, et al. Association between the UGT1A1*28 allele, bilirubin levels, and coronary heart disease in the Framingham Heart Study. Circulation. 2006. 10.1161/CIRCULATIONAHA.106.633206 17000907

[6] Wang J, Guo P, Gao Z, Zhou B, Ren L, Chen Y. Elevated bilirubin levels and risk of developing chronic kidney disease: a dose–response meta ‑ analysis and systematic review of cohort studies. Int Urol Nephrol. Springer Netherlands; 2018;50(2):275–87. doi: 10.1007/s11255-017-1675-y 28808864

[7] Yang T, Lin Y, Lin Y, Huang C, Chen H, Wu M. Total Bilirubin in Prognosis for Mortality in End-Stage Renal Disease Patients on Peritoneal Dialysis Therapy. 2017;1–7. 10.1161/JAHA.117.007507 PMC5779053 29275374

[8] Boon A, Bulmer AC, Coombes JS, Fassett RG. Novel Therapeutics in Renal Diseases Circulating bilirubin and defense against kidney disease and cardiovascular mortality: mechanisms contributing to protection in clinical investigations. 2019;2. 10.1152/ajprenal.00039.2014 24761005

[9] Hosoya T, Ohno I, Nomura S, Hisatome I, Uchida S, Fujimori S, et al. Effects of topiroxostat on the serum urate levels and urinary albumin excretion in hyperuricemic stage 3 chronic kidney disease patients with or without gout. Clin Exp Nephrol. 2014. 10.1007/s10157-014-0935-8 PMC4271138 24448692

[10] Hamamoto S, Kaneto H, Kamei S, Shimoda M, Tawaramoto K, Kanda-Kimura Y, et al. Low bilirubin levels are an independent risk factor for diabetic retinopathy and nephropathy in Japanese patients with type 2 diabetes [Internet]. Vol. 41, Diabetes and Metabolism. Elsevier Masson SAS; 2015 [cited 2020 Jul 24]. p. 429–31. https://pubmed.ncbi.nlm.nih.gov/26054296/. 10.1016/j.diabet.2015.05.003 26054296

[11] Singer S, Pilpel N, Pinhas-Hamiel O. Gilbert syndrome in patients with type 1 diabetes—Prevalence, glycemic control, and microalbuminuria. Pediatr Diabetes [Internet]. Blackwell Publishing Ltd; 2017 Dec 1 [cited 2020 Jul 24];18(8):803–7. Available from: https://pubmed.ncbi.nlm.nih.gov/28093842/. doi: 10.1111/pedi.12488 28093842

[12] Wang J, Li Y, Han X, Hu H, Wang F, Yu C, et al. Association between serum bilirubin levels and decline in estimated glomerular filtration rate among patients with type 2 diabetes. J Diabetes Complications [Internet]. Elsevier Inc.; 2016 Sep 1 [cited 2020 Jul 24];30(7):1255–60. Available from: https://pubmed.ncbi.nlm.nih.gov/27288202/. doi: 10.1016/j.jdiacomp.2016.05.013 27288202

[13] Vuruşkan E, Saraçoǧlu E. Bilirubin Levels are Associated with Contrast-Induced Nephropathy in Peripheral Artery Disease. Angiology [Internet]. SAGE Publications Inc.; 2017 Sep 1 [cited 2020 Jul 24];68(8):728–33. Available from: https://pubmed.ncbi.nlm.nih.gov/27852844/. doi: 10.1177/0003319716679340 27852844

[14] Zhou X, Jiang J, Ren W, Fei Y, Peng L, Jiang J, et al. Related factors of diabetic nephropathy in patients with type 1 diabetes mellitus. Natl Med J China [Internet]. Chinese Medical Association; 2018 Aug 14 [cited 2020 Jul 24];98(30):2403–6. Available from: https://pubmed.ncbi.nlm.nih.gov/30138984/. doi: 10.3760/cma.j.issn.0376-2491.2018.30.007 30138984

[15] Ahn KH, Kim SS, Kim WJ, Kim JH, Nam YJ, Park S Bin, et al. Low serum bilirubin level predicts the development of chronic kidney disease in patients with type 2 diabetes mellitus. Korean J Intern Med [Internet]. Korean Association of Internal Medicine; 2017 [cited 2020 Jul 24];32(5):875–82. Available from: https://pubmed.ncbi.nlm.nih.gov/28560862/28560862 10.3904/kjim.2015.153 PMC5583441

[16] Sakoh T, Nakayama M, Tanaka S, Yoshitomi R, Ura Y, Nishimoto H, et al. Association of serum total bilirubin with renal outcome in Japanese patients with stages 3–5 chronic kidney disease. Metabolism [Internet]. W.B. Saunders; 2015 Sep 1 [cited 2020 Jul 24];64(9):1096–102. Available from: https://pubmed.ncbi.nlm.nih.gov/26142826/. doi: 10.1016/j.metabol.2015.06.006 26142826

[17] Zhu B, Wu X, Bi Y, Yang Y. Effect of bilirubin concentration on the risk of diabetic complications: A meta-analysis of epidemiologic studies. Sci Rep [Internet]. Nature Publishing Group; 2017 Jan 30 [cited 2020 Jul 24];7. Available from: https://pubmed.ncbi.nlm.nih.gov/28134328/. doi: 10.1038/srep41681 PMC5278382 28134328

[18] Moolchandani K, Priyadarssini M, Rajappa M, Parameswaran S, Revathy G. Serum bilirubin: a simple routine surrogate marker of the progression of chronic kidney disease. Br J Biomed Sci [Internet]. Taylor and Francis Ltd.; 2016 Oct 1 [cited 2020 Jul 24];73(4):188–93. Available from: https://pubmed.ncbi.nlm.nih.gov/27231984/. 10.1080/09674845.2016.1182674 27231984

[19] Hughes JT, Barzi F, Hoy WE, Jones GRD, Rathnayake G, Majoni SW, et al. Bilirubin concentration is positively associated with haemoglobin concentration and inversely associated with albumin to creatinine ratio among Indigenous Australians: eGFR Study. Clin Biochem [Internet]. Elsevier; 2017;50(18):1040–7. Available from: doi: 10.1016/j.clinbiochem.2017.08.011 28834701

[20] Li X, Zhang L, Chen H, Guo K, Yu H, Zhou J, et al. Relationship between serum bilirubin concentrations and diabetic nephropathy in Shanghai Han’s patients with type 1 diabetes mellitus. BMC Nephrol [Internet]. BioMed Central Ltd.; 2017 Mar 31 [cited 2020 Jul 24];18(1). Available from: https://pubmed.ncbi.nlm.nih.gov/28363276/. doi: 10.1186/s12882-017-0531-8 PMC5376273 28363276

[21] Ren Y, Gao L, Guo X, Huo X, Lu J, Li J, et al. Interactive effect of serum uric acid and total bilirubin for micro-vascular disease of type 2 diabetes in China. J Diabetes Complications [Internet]. Elsevier Inc.; 2018 Nov 1 [cited 2020 Jul 24];32(11):1000–5. Available from: https://pubmed.ncbi.nlm.nih.gov/30224234/. doi: 10.1016/j.jdiacomp.2018.09.002 30224234

[22] Nishimura T, Tanaka M, Sekioka R, Itoh H. Serum bilirubin concentration is associated with EGFR and urinary albumin excretion in patients with type 1 diabetes mellitus. J Diabetes Complications [Internet]. Elsevier Inc.; 2015 Nov 1 [cited 2020 Jul 24];29(8):1223–7. Available from: https://pubmed.ncbi.nlm.nih.gov/26234498/. doi: 10.1016/j.jdiacomp.2015.07.007 26234498

[23] Mortada I. Hyperbilirubinemia, Hypertension, and CKD: the Links [Internet]. Vol. 19, Current Hypertension Reports. Current Medicine Group LLC 1; 2017 [cited 2020 Jul 24]. https://pubmed.ncbi.nlm.nih.gov/28597405/. 10.1007/s11906-017-0756-8 28597405

[24] Liu Y, Li M, Song Y, Liu X, Zhao J, Deng B, et al. Association of serum bilirubin with renal outcomes in Han Chinese patients with chronic kidney disease. Clin Chim Acta [Internet]. Elsevier B.V.; 2018 May 1 [cited 2020 Jul 24];480:9–16. Available from: https://pubmed.ncbi.nlm.nih.gov/29408172/. doi: 10.1016/j.cca.2018.01.041 29408172

[25] Mashitani T, Hayashino Y, Okamura S, Tsujii S, Ishii H. Correlations between serum bilirubin levels and diabetic nephropathy progression among japanese type 2 diabetic patients: A prospective cohort study (diabetes distress and care registry at tenri [DDCRT 5]). Diabetes Care. 2014. 10.2337/dc13-0407 24009299

[26] Park S, Kim DH, Hwang JH, Kim YC, Kim JH, Lim CS, et al. Elevated bilirubin levels are associated with a better renal prognosis and ameliorate kidney fibrosis. PLoS One. 2017. doi: 10.1371/journal.pone.0172434 28225832 PMC5321406

[27] Fukui M, Tanaka M, Shiraishi E, Harusato I, Hosoda H, Asano M, et al. Relationship between serum bilirubin and albuminuria in patients with type 2 diabetes. Kidney Int. 2008. doi: 10.1038/ki.2008.398 18854849

[28] Li J, Liu D, Liu Z. Serum Total Bilirubin and Progression of Chronic Kidney Disease and Mortality: A Systematic Review and Meta-Analysis. Front Med. 2021;7(January):1–10. doi: 10.3389/fmed.2020.00549 33569386 PMC7868400

[29] Braunwald E, Steg PG, Davidson J, Hirshberg B, Ohman P, Frederich R, et al. Saxagliptin and Cardiovascular Outcomes in Patients with Type 2 Diabetes Mellitus. N Engl J Med. 2013;369(14):1317–26. doi: 10.1056/NEJMoa1307684 23992601

[30] Nissen F, Quint JK, Wilkinson S, Mullerova H, Smeeth L, Douglas IJ. Validation of asthma recording in electronic health records: protocol for a systematic review. 2017;1–5. 10.1136/bmjopen-2016-014694 PMC5729974 28554919

[31] Herrett E, Gallagher AM, Bhaskaran K, Forbes H, Mathur R, Van Staa T, et al. Data Resource Profile: Clinical Practice Research Datalink (CPRD). 2015;(June):827–36. 10.1093/ije/dyv098 PMC4521131 26050254

[32] Wolf A, Dedman D, Campbell J, Booth H, Lunn D, Chapman J, et al. Data resource profile: Clinical Practice Research Datalink (CPRD) Aurum. 2019;(March). 10.1093/ije/dyz034 PMC6929522 30859197

[33] the Government Digital Service. UK Read Code [Internet]. [cited 2022 Mar 29]. https://data.gov.uk/dataset/f262aa32-9c4e-44f1-99eb-4900deada7a4/uk-read-code.

[34] Levey AS, Stevens LA, Schmid CH, Zhang YL, Iii AFC, Feldman HI, et al. A New Equation to Estimate Glomerular Filtration Rate. 2006. 10.7326/0003-4819-150-9-200905050-00006 PMC2763564 19414839

[35] Austin PC, Stuart EA. Moving towards best practice when using inverse probability of treatment weighting (IPTW) using the propensity score to estimate causal treatment effects in observational studies. 2015;(April). 10.1002/sim.6607 PMC4626409 26238958

[36] Pearl J, Robins JM, Pearl J, Robins JM. Causal Diagrams for Epidemiologic Research. 2018;10(1):37–48. 9888278

[37] ROBINS TJVAJM. Minimal sufficient causation and directed acyclic graphs. Tha Ann Stat. 2009;37(3):1437–65.

[38] Dsc DGA, Moons KGM. when not to use the missing-indicator method for analysis. 2012;184(11):1265–9. 10.1503/cmaj.110977 PMC3414599 22371511

[39] Meyer B, Scholtz H, Schall R, Muller F, Hundt H, Maree J. The effect of fasting on total serum bilirubin concentrations. Br J Clin Pharmacol. 1995. doi: 10.1111/j.1365-2125.1995.tb04424.x 7742155 PMC1364954

[40] Pocock SJ, Ashby D, Shaper AG, Walker M, Broughton PMG. Diurnal variations in serum biochemical and haematological measurements. J Clin Pathol. 1989. doi: 10.1136/jcp.42.2.172 2921359 PMC1141821

[41] Riphagen IJ, Deetman PE, Bakker SJL, Navis G, Cooper ME, Lewis JB, et al. Bilirubin and progression of nephropathy in type 2 diabetes: A post hoc analysis of RENAAL with independent replication in IDNT. Vol. 63, Diabetes. 2014. 10.2337/db13-1652 24677717

[42] US Food and Drug Administration. Enrichment strategies for clinical trials to support determination of effectiveness of human drugs and biological products guidance for industry. 2019.

[43] Hull TD, Agarwal A. Bilirubin: A potential biomarker and therapeutic target for diabetic nephropathy. Diabetes. 2014;63(8):2613–6. doi: 10.2337/db14-0691 25060893 PMC4113062

