# Supplementary material for: Bilirubin levels and kidney function decline: An analysis of clinical trial and real world data
Source: PLoS One. 2022 Jun 21;17(6):e0269970. doi: 10.1371/journal.pone.0269970 (PMC9212140; doi:10.1371/journal.pone.0269970)
Supplement: S1 File — (DOCX) [file pone.0269970.s001.docx]

INDEPENDENT SCIENTIFIC ADVISORY COMMITTEE (ISAC) PROTOCOL APPLICATION FORM

PART 1: APPLICATION FORM

***IMPORTANT***

**Both parts of this application must be completed in accordance with the guidance note ‘Completion of the ISAC Protocol Application Form’, which can be found on the CPRD website** [**cprd.com/research-applications**](https://cprd.com/research-applications)

| FOR ISAC USE ONLY | |
| --- | --- |
| **Protocol No. – 19_144** | **Submission date -** |

| GENERAL INFORMATION ABOUT THE PROPOSED RESEARCH STUDY |
| --- |
| Study Title (Max. 255 characters) Association of serum bilirubin level with renal outcomes in patients with type 2 diabetes and patients with essential hypertension. |
| **Research Area** (place ‘X’ in all boxes that apply) |
| \| Drug Safety \|  \| Economics \|  \| \| --- \| --- \| --- \| --- \| \| Drug Utilisation \|  \| Pharmacoeconomics \|  \| \| Drug Effectiveness \|  \| Pharmacoepidemiology \|  \| \| Disease Epidemiology \| x \| Methodological \|  \| \| Health Services Delivery \|  \|  \|  \| |
| Chief Investigator  \| Title: \| Dr \| \| --- \| --- \| \| Full name: \| Dinko Rekić \| \| Job title: \| Senior Clinical Pharmacometrician \| \| Affiliation/organisation: \| AstraZeneca \| \| Email address: \| dinko.rekic@astrazeneca.com \| \| CV Number (if applicable): \| 347_19 \| |
| Corresponding Applicant  \| Title: \| Dr \| \| --- \| --- \| \| Full name: \| Yasunori Aoki \| \| Job title: \| Post Doctoral Research Fellow \| \| Affiliation/organisation: \| AstraZeneca \| \| Email address: \| Yasunori.aoki1@astrazeneca.com \| \| CV Number (if applicable): \| 348_19 \| |
| List of all investigators/collaborators  \| Title: \| Dr \| \| --- \| --- \| \| Full name: \| Claudia Cabrera \| \| Job title: \| Director Epidemiology \| \| Affiliation/organisation: \| AstraZeneca, Karolinska Institute \| \| Email address: \| Claudia.S.Cabrera@astrazeneca.com \| \| CV Number (if applicable): \| 133_15CES \| \| Will this person be analysing the data? (Y/N) \| N \|  \| Title: \| Dr \| \| --- \| --- \| \| Full name: \| Peter Greasley \| \| Job title: \| Director Physician \| \| Affiliation/organisation: \| AstraZeneca \| \| Email address: \| Peter.Greasley@astrazeneca.com \| \| CV Number (if applicable): \| 349_19 \| \| Will this person be analysing the data? (Y/N) \| N \| |
| Experience/expertise available List below the member(s) of the research team who have experience with CPRD data.   \| **Name:** \| **Protocol Number/s:** \| \| --- \| --- \| \| Claudia Cabrera \| 17_059; 15_216R; 18_080 \| \|  \|  \| \|  \|  \|   List below the member(s) of the research team who have statistical expertise.   \| **Name(s):** \|  \| \| --- \| --- \| \| Yasunori Aoki \| \| \| Dinko Rekić \| \| \|  \| \|   List below the member(s) of the research team who have experience of handling large datasets (greater than 1 million records).   \| **Name(s):** \|  \| \| --- \| --- \| \| Yasunori Aoki \| \| \|  \| \| \|  \| \|   List below the member(s) of the research team, or supporting the research team, who have experience of practicing in UK primary care.   \| **Name(s):** \|  \| \| --- \| --- \| \|  \| \| \|  \| \| \|  \| \| |
| ACCESS TO THE DATA |
| Sponsor of the study  \| Institution/Organisation: \| AstraZeneca R&D Mölndal \| \| --- \| --- \| \| Address: \| Pepparedsleden 1, 431 50 Mölndal, Sweden \| |
| Funding source for the study  \| Same as Sponsor? \| Yes \| x \| No \|  \|  \| \| --- \| --- \| --- \| --- \| --- \| --- \| \| Institution/Organisation: \|  \| \| \| \| \| \| Address: \|  \| \| \| \| \| |
| Institution conducting the research  \| Same as Sponsor? \| Yes \| x \| No \|  \|  \| \| --- \| --- \| --- \| --- \| --- \| --- \| \| Institution/Organisation: \|  \| \| \| \| \| \| Address: \|  \| \| \| \| \| |
| Data Access Arrangements Indicate with an ‘**X**’ the method that will be used to access the data for this study:   \| Study-specific Dataset Agreement \|  \| \| --- \| --- \|  \| Institutional Multi-study Licence \| x \|  \| \| --- \| --- \| --- \| \| Institution Name \| AstraZeneca UK Limited \| \| \| Institution Address \| 1 Francis Crick Avenue, Cambridge Biomedical Campus, Cambridge CB2 0AA, England \| \|   Will the dataset be extracted by CPRD?   \| Yes \|  \| No \| x \| \| --- \| --- \| --- \| --- \|   If yes, provide the reference number: |
| 1. **Data Processor(s):**  \| Processing \| x \|  \| \| --- \| --- \| --- \| \| Accessing \| x \| \| Storing \| x \| \| Processing area (UK/EEA/Worldwide) \| \| Worldwide \| \| Organisation name \| \| AstraZeneca \| \| Organisation address \| \| AstraZeneca Philadelphia Data Center. 1500 Spring Garden Street, 3rd Floor Phase 3C, Philadelphia, PA 19310. (Netezza and SAS Grid) \|  \| Processing \|  \|  \| \| --- \| --- \| --- \| \| Accessing \|  \| \| Storing \|  \| \| Processing area (UK/EEA/Worldwide) \| \|  \| \| Organisation name \| \|  \| \| Organisation address \| \|  \| |
| INFORMATION ON DATA |
| Primary care data (place ‘X’ in all boxes that apply)  \| CPRD GOLD \| x \| CPRD Aurum \|  \| \| --- \| --- \| --- \| --- \|   **X** |
| Please select any linked data or data products being requested **Patient Level Data** (place ‘**X**’ in all boxes that apply) |
| \| ONS Death Registration Data \|  \| CPRD Mother Baby Link \|  \| \| --- \| --- \| --- \| --- \| \| HES Admitted Patient Care \|  \| Pregnancy Register \|  \| \| HES Outpatient \|  \| NCRAS (National Cancer Registration and Analysis Service) Cancer Registration Data \|  \| \| HES Accident and Emergency \|  \| NCRAS Cancer Patient Experience Survey (CPES) data \|  \| \| HES Diagnostic Imaging Dataset \|  \| NCRAS Systemic Anti-Cancer Treatment (SACT) data \|  \| \| HES PROMS (Patient Reported Outcomes Measure) \|  \| NCRAS National Radiotherapy Dataset (RTDS) data \|  \| \|  \|  \| Mental Health Services Data Set (MHDS) \|  \| |
| **Area Level Data** (place ‘**X**’ in all boxes that apply)   \| **Practice level (UK)** \|  \| **Patient level (England only)** \|  \| \| --- \| --- \| --- \| --- \| \| Practice Level Index of Multiple Deprivation (Standard) \|  \| Patient Level Index of Multiple Deprivation \|  \| \| Practice Level Index of Multiple Deprivation (Non-standard) \|  \| Patient Level Townsend Score \|  \| \| Practice Level Index of Multiple Deprivation Domains (Non-standard) \|  \|  \|  \| \| Practice Level Carstairs Index for 2011 Census (Excluding Northern Ireland) (Standard) \|  \|  \|  \| \| 2011 Rural-Urban Classification at LSOA level (Non-standard) \|  \|  \|  \|   Reference number (where applicable): |
| Are you requesting linkage to a dataset not listed above?  \| Yes \|  \| No \| **x** \| \| --- \| --- \| --- \| --- \|   If yes, provide the reference number: |
| Does any person named in this application already have access to any of these data in a patient identifiable form, or associated with an identifiable patient index?  \| Yes \|  \| No \| **x** \| \| --- \| --- \| --- \| --- \|   If yes, provide further details: |
| VALIDATION/VERIFICATION |
| Does this protocol describe an observational study using purely CPRD data?  \| Yes \| **x** \| No \|  \| \| --- \| --- \| --- \| --- \| |
| Does this protocol involve requesting any additional information from GPs, or contact with patients?  \| Yes \|  \| No \| **x** \| \| --- \| --- \| --- \| --- \|   If yes, provide the reference number: |

**PART 2: PROTOCOL INFORMATION**

| **Applicants must complete all sections listed below**  **Sections which do not apply should be completed as ‘*Not Applicable’* and justification provided** |
| --- |
| Study Title (Max. 255 characters) Association of serum bilirubin level with renal outcomes in patients with type 2 diabetes and patients with essential hypertension. |
| Lay Summary (Max. 250 words) Bilirubin is a substance naturally produced by the body when the red blood cells get old and break down. Its level in the body varies to a large degree between people. It has been proposed that the anti-oxidative effect of bilirubin may protect us from kidney diseases. Majority of published studies are supporting this protective effect of bilirubin while some researches are still sceptical. We wish to conduct high-quality analyses on high-quality data created from the CPRD GOLD population to provide supporting evidence to this scientific question. We propose to divide patients in the CPRD dataset into those with high and low-level bilirubin and compare how their kidney function changes over time.  Patients with type 2 diabetes or with high blood pressure are at additional risk of losing kidney function. When kidney function is lost, the body cannot filter out the waste products in the blood effectively which leads to life threatening adverse events such as a heart attack. If we can show that bilirubin protects kidney function, then we can potentially predict the patients who are at higher risk of kidney disease based on bilirubin level. This will allow doctors to more closely monitor the patients for renal disease and provide better treatment. |
| Technical Summary (Max. 300 words) Recent investigations and meta-analyses indicate a strong association between bilirubin and renal outcomes. We wish to conduct a retrospective study on CPRD GOLD dataset to investigate these associations in a real-world setting. Based on our preliminary feasibility count, we believe the cohorts retrospectively built using CPRD GOLD dataset will be at least one order of magnitude larger than the analyses that are currently available. Also, we should be able to adjust critical confounders such as haemoglobin level and smoking status that was previously not done or lead to inconclusive results.  We will retrospectively build two cohorts with known risk factors for CKD: type 2 diabetes or hypertension. We will follow the patients from the date of diagnosis of either of these morbidities to one of the events indicating the degradation of renal function (eGFR decrease, serum creatinine increase, albuminuria, or proteinuria). We will fit the Cox proportional hazard models with inverse probability weighting to estimate the adjusted hazard ratio between the patients with elevated bilirubin vs. normal levels.  We are aware that as this will only be the observational study and we will not be able to conclude the causality of bilirubin levels to CKD progression even if we can show an association. However, with careful analyses based on richer dataset than what is currently available, this analysis may increase our understanding of the association between bilirubin and CKD outcomes and guide further investigations. |
| Outcomes to be Measured  1. Time to eGFR CKDEPI 30% decrease from the baseline; 2. Time to first observation of proteinuria; 3. Time to first observation of albuminuria. |
| Objectives, Specific Aims and Rationale Overall Primary Objective:  1a) To investigate the risk of worsening of renal function using eGFR (using CKD EPI formula) in relation to bilirubin levels in type2 diabetic patients, in a real-world setting.  1b) To investigate the risk of worsening of renal function using eGFR in relation to bilirubin levels in hypertensive patients, in a real-world setting.  Secondary objectives:  2a) To investigate the risk of worsening of renal function using urine albumin creatinine ratio (UACR) in relation to bilirubin levels in type2 diabetic patients, in a real-world setting.  2b) To investigate the risk of worsening of renal function using UACR in relation to bilirubin levels in hypertensive patients, in a real-world setting.  Exploratory objectives:  3a) To investigate the risk of worsening of renal function using amount of protein in urine in relation to bilirubin levels in type2 diabetic patients, in a real-world setting.  3b) To investigate the risk of worsening of renal function using amount of protein in urine in relation to bilirubin levels in hypertensive patients, in a real-world setting.  Specific aims:   \| **Primary objectives**  1a) To investigate the risk of worsening of renal function using eGFR in relation to bilirubin levels in type2 diabetic patients, in a real-world setting.  1b) To investigate the risk of worsening of renal function using eGFR in relation to bilirubin levels in hypertensive patients, in a real-world setting. \| **Outcome measure**  Time to first of first two consecutive measurements of eGFR that are 30 percent less than the eGFR at baseline. \| **Hypothesis tested**  1a) Naturally elevated serum bilirubin level of type2 diabetic patients is associated with slower degradation of the renal function when renal function is measured using eGFR.  1b) Naturally elevated serum bilirubin level of hypertensive patients is associated with slower degradation of the renal function when renal function is measured using eGFR. \| \| --- \| --- \| --- \| \| **Secondary objective**  2a) To investigate the risk of worsening of renal function using urine UACR in relation to bilirubin levels in type2 diabetic patients, in a real-world setting.  2b) To investigate the risk of worsening of renal function using UACR in relation to bilirubin levels in hypertensive patients, in a real-world setting. \| **Outcome measure**  Time to first observation of albuminuria (UACR >30mg/g) during the follow up period. \| **Hypothesis tested**  2a) Naturally elevated serum bilirubin level of type2 diabetic patients is associated with slower degradation of the renal function when renal function evaluated by UACR.  2b) Naturally elevated serum bilirubin level of hypertensive patients is associated with slower degradation of the renal function when renal function evaluated by UACR. \| \| **Exploratory objective**  3a) To investigate the risk of worsening of renal function using amount of protein in urine in relation to bilirubin levels in type2 diabetic patients, in a real-world setting.  3b) To investigate the risk of worsening of renal function using amount of protein in urine in relation to bilirubin levels in hypertensive patients, in a real-world setting. \| **Outcome measure**  Time to first observation of proteinuria during the follow up period. \| **Hypothesis tested**  3a) Naturally elevated serum bilirubin level of type2 diabetic patients is associated with slower degradation of the renal function when renal function evaluated by proteinuria.  3b) Naturally elevated serum bilirubin level of hypertensive patients is associated with slower degradation of the renal function when renal function evaluated by proteinuria. \|   **Rational:**  Recent investigations as well as meta-analyses indicate a strong association between serum bilirubin concentrations and renal outcomes. This association has led us to hypothesis that elevated bilirubin level is associated with slower degradation of kidney function. There are still discussions on this association because the previous investigations lack diversity in the patient population and did not adjust for the key confounders. We wish to conduct a retrospective study in a real-world setting to support or dispute this hypothesis. This study will provide the medical community extra piece of evidence for or against the bilirubin as a potential predictive marker for CKD progression as well as the target for preventative treatment option. |
| Study Background Bilirubin is the most powerful endogenous lipophilic anti-oxidant and is eliminated through glucuronidation by uridine diphosphate glucuronosyltransferase 1 family, polypeptide A1 (UGT1A1). It has been hypothesized that naturally elevated level of bilirubin has a protective effect against renal outcomes. The mechanism of action is proposed to be anti-oxidative, with effects on inflammation, endothelial function, dyslipidaemia, and platelet hyperactivity (1,2). Recent investigations indicate strong associations between naturally elevated bilirubin concentrations and positive renal outcome in diabetic and or hypertensive patients (3,4,5,6).  Most of the currently available studies (3,5,6) were conducted in Asia. It is therefore unclear if the results would be similar in non-Asian populations. Wagner et al. (7) showed that the natural bilirubin level is usually higher in Asians than Europeans, hence it is plausible that the findings based on Asian population (in 3,5,6) may not extend to the European population. To best of our knowledge, Riphagen et al. conducted the only study outside of Asia, showing a positive effect of bilirubin on a composite endpoint consisting of serum creatinine doubling or end stage renal disease (4).  All of these studies consisted of approximately 5000 subjects or less and may not have been able to account for critical confounders such as hemoglobin or smoking status which we hope to capture through a real-world study.  Prior studies focused on two patient populations at risk of CKD, diabetics or hypertensive patients. However, because different studies used different endpoints and slightly different methodologies, it is difficult to make direct comparisons on the potential effect of bilirubin in the two populations. Thus, by creating two cohorts, diabetic cohort and the hypertensive cohort from the same database and applying the identical analysis method we may be able to make direct comparisons on the effect of bilirubin on CKD in these two populations. |
| Study Type Observational study, part descriptive and part analytical. |
| Study Design This will be a retrospective open cohort study.  The first cohort (CH1) will be the population with a diagnosis date of type 2 diabetes. The second cohort (CH2) will be the population with a diagnosis date of essential hypertension. |
| Feasibility counts The number of patients in each cohort and number of patients who will be included in the analysis of each objective is listed below (the feasibility count is conducted using the CPRD data up to July 2018) please see Appendices 1 and 2 for exclusion diagram, please see Section L for inclusion exclusion criteria as well as objective specific exclusion criteria:   \|  \| CH1 (Type2 Diabetic patients) \| CH2 (Hypertensive patients) \| \| --- \| --- \| --- \| \| Number of subjects in Cohort \| 68 217 \| 85 409 \| \| Number of subjects for the analyses of the primary objectives 1a) and 1b) (endpoint: eGFR 30% decrease) \| 66 509 \| 81 025 \| \| Number of subjects for the analyses of the exploratory objectives 3a) and 3b) (endpoint: albuminuria) \| 45 168 \| 79 975 \| \| Number of subjects for the analyses of the secondary objectives 2a) and 2b) (endpoint: proteinuria) \| 66 317 \| 84 335 \| |
| Sample size considerations Based on our analyses on in-house randomised control clinical trial (RCT) data of type2 diabetic patients, we guess the hazard ratio for the primary endpoint to be 0.8 for the above median bilirubin level subgroup, in addition, we assume 5% type I error is tolerable and require 80% power. Based on these assumptions, using the formula derived by Schoenfeld (8), using the implementation available at <http://www.sample-size.net/sample-size-survival-analysis/>, we obtain that we need 631 events.  Using internal AZ data from a recent RCT we calculated our sample size estimate. In that in-house dataset, the event rate was approximately 0.047 per year per person. Hence assuming similar event rates, we require 13 500 person-year to achieve this power. According to our feasibility count, each cohort will include over 60 000 patients with a median follow up period of approximately 5 years (i.e., most likely over 300 000 person-year), hence we believe we have enough power to reliably conduct this study. |
| Planned use of linked data (if applicable): We will not use linked data. |
| Definition of the Study population This study will examine two patient cohorts who are at risk of CKD, diabetic and hypertensive patients. Eligible patients must have information recorded at least 12 months prior to the study index date and have up to standard (UTS). The study population will include patient records from January 2005 to January 2019.  **Inclusion criteria**  Cohort 1 (CH1) will include type 2 diabetes mellitus (T2D) patients along with any one of the following criteria (see Appendix 1 for the inclusion exclusion flow chart):   - Newly diagnosed T2D (MEDCODE 758 : Type 2 diabetes mellitus) - 2 prescriptions of diabetic medication (See Appendix 3 for the list of all drugs) - Hemoglobin A1c (HbA1c) value greater than 6.5   Cohort 2 (CH2) will include essential hypertension (EHT) patients along with the following criteria (see Appendix 2 for the inclusion exclusion flow chart):   - Newly diagnosed essential hypertension (see Appendix 4 for list of Read codes)   Time related variable including the index date and the follow-up is described in the following figure:  **Rolling index date**  (The date first diagnosed with DM2 or HT**)**  Pre-index period  1 years  Acute disease period  2 years  Follow up period  (Median follow up 5 years, minimum follow up 1 year)  **End of follow up**  (Either the date patient has transferred out from the practice  Or the last date of the record in the dataset)  **CH1**: no DM2 diagnosis nor related medication during this period **CH2**: no HT diagnosis nor DM2 diagnosis nor related medication during this period  Baseline is characterized based on the observation in these periods  January 2005  January 2019  **Exclusion criteria**  We will exclude the following patients based on the Read code during acute disease period (2 years after the rolling index date):   - Liver dysfunction including drug induced liver injury (diagnosis with Read code listed in Appendix 5 and/or Aspartate transaminase (AST) and alanine transaminase (ALT) levels outside of the normal range (AST: 8-48U/L, ALT: 7-55U/L) - Anemia (hemoglobin level below cutoff, female:120 male:130 g/L, or with the clinical annotation of primary or secondary anemia with the Read code listed in Appendix 6) - Human immunodeficiency virus (HIV) infection and or on HIV related medications (see Appendix 7). - All malignancies and or on medications related to the treatment for the malignancies (see Appendix 8). - Age < 40 (to exclude type 1 diabetic patients). - Any user of UGT1a1 inhibitors (see Appendix 9) during the acute disease period. - < 1 year of clinical records in the database before the index date - Pregnant women - Patients with clinical record of alcohol dependencies. - Any patients with less than two bilirubin measurements during the acute disease period.   **Objective-specific exclusion criteria**  For each endpoint, we impose the following additional exclusion criterion:   - For objectives 1a) and 1b) with the endpoint “First two consecutive measurements of eGFR that are 30 percent less than the last eGFR record during the acute disease period”, we will exclude patients without eGFR records during the acute disease period (2 years after the rolling index date). - For objectives 2a) and 2b) with the endpoint “First observation of albuminuria (UACR >30mg/g) during the follow up period.”, we will exclude patients with recorded UACR more than 30mg/g during the acute disease period. - For objectives 3a) and 3b) with the endpoint “First observation of proteinuria during the follow up period, we will exclude the patients with the recorded proteinuria (protein dipstick test result +, ++, or +++) during the acute disease period. |
| Selection of comparison group(s) or controls We will stratify the patients based on the population median of the patient mean baseline serum bilirubin levels from the tests table under ENTTYPE 158. We consider the patients with less than or equal to the median serum bilirubin level to be the control group, and patients with greater than the median serum bilirubin level to be the exposure group.  The serum bilirubin levels are determined by the average of all the available measurements during the acute disease period (2 years after the rolling index date). We require at least two bilirubin measurements to be used to obtain an average estimate (this will account for the variability of bilirubin measurements). |
| Exposures, Outcomes and Covariates **Outcome:**  Outcomes will include the following events:  Primary objective: eGFR decline  1) First two consecutive measurements of eGFR that are 30 percent less than the last eGFR record during the acute disease period.  eGFR will be calculated using CKD EPI formula based on the recorded serum creatinine measurements in tests table ENTTYPE 165.  Secondary objective: Proteinuria  2) First observation of the proteinuria  Proteinuria is defined as the protein dipstick test results either +, ++ or +++, recorded in the tests table under ENTTYPE 431.  Exploratory objective: Albuminuria  3) First observation of the albuminuria  Albuminuria is defined as the recorded UACR measure in test table under ENTTYPE 469 greater than 30mg/g.  **Covariates:**  We will use all the available covariates (see Appendix 10) to calculate the propensity score that we will use for the inverse probability weighting of the Cox proportional hazard model.  We will include all the available covariates (see Appendix 10) in the baseline table.  We will include the following selected covariates in the multivariate Cox proportional hazard model:   - Age, Sex, Weight, BMI, Baseline eGFR, Baseline UACR, Baseline protein dipstick result, Smoking status, Serum Haemoglobin level, HDL cholesterol, LDL cholesterol - Use of the following drugs: ACE inhibitor, Statin, Diuretics, Opioids, Potassium-sparing diuretics |
| Data/ Statistical Analysis Baseline characterization  The baseline characteristics will be described by the exposure groups (high and low bilirubin level at baseline). Summary statistics such as counts, average, standard deviation, median, and inter-quantile range will be tabulated.  Formal analysis of individual cohort  Multivariate Cox’s proportional hazard model with stabilized inverse probability weighting scheme (9,10) will be used to calculate the Hazard ratios (HR) with 95% confidence interval for the outcomes (worsening of eGFR, proteinuria, and albuminuria).  Sensitivity analysis  We will test the robustness of the results from the formal analysis by testing the sensitivity to   - the use of inverse probability weighting. (We will repeat the formal analysis without inverse probability weighting and compare the HR) - the cut-off value for the serum bilirubin level for dividing control and exposure group. (We will repeat the formal analysis with different bilirubin cut-offs and compare the HR) - the subpopulation of the patients. Subgroups will be made based on the following covariates: Age, Sex, smoking status, Baseline eGFR, Baseline UACR, Baseline protein dipstick result, Use of ACE inhibitor, Statin, Diuretics, Opioids, Potassium-sparing diuretics. (We will conduct subgroup analysis with test of interaction between the subgroup and exposure-control groups)   In addition, to investigate the possible bias introduced by the fact we are including only the patients with two or more bilirubin measurements at the baseline, we will repeat the above analyses but compare the patient population with baseline bilirubin measurement v.s. no baseline bilirubin measurements.  Additional analysis   - We will characterize bilirubin as continuous variable to understand the medically important nonlinear relationship between bilirubin concentration and outcomes. - We will build mixed effect model on eGFR and test statistical significance of bilirubin level as a covariate using likelihood ratio test to better understand the time course variation of eGFR in relation to bilirubin level. |
| Plan for addressing confounding We will use inverse-probability weighting to address the confounding. |
| Plans for addressing missing data We will use missing indicator method to handle missing data. |
| Patient or user group involvement (if applicable) No patient/user groups will be engaged in any way.  This is an explorative study to better understand the impact of bilirubin on a population level. Furthermore, no patients will be contacted we will only use CPRD retrospective data and we do not have any plans to assess quality of life. This is an exploratory study which will further our scientific knowledge about the impact of bilirubin on renal outcomes and potentially support the design of future clinical trials. |
| Plans for disseminating and communicating study results, including the presence or absence of any restrictions on the extent and timing of publication We plan to communicate the study result regardless of either positive or negative result though academic publication in a medical journal. As this is an academic project for the postdoctoral research fellow (YA) and he is hired for this project with time limiting contract (ending September 2020, extendable up to September 2021) hence the manuscript needs to be submitted to the journal before the end of his contract.  **Conflict of interest statement:**  All the investigators are employed by AstraZeneca, however, this study does not focus on AstraZeneca or any other medicines. |
| Limitations of the study design, data sources, and analytic methods As bilirubin is endogenous substance, the patients are exposed to them throughout their entire life hence the development of type 2 diabetes or hypertension can be affected by bilirubin.  Also, it is known that the natural level of bilirubin depends on the race, gender and other patient characteristics. Hence there will be a bias in baseline characteristics for exposure groups. Furthermore, as our primary analysis compares bilirubin levels it is only possible to conduct these analyses among subjects with bilirubin measurements and baseline measurements of each outcome. This may impact generalizability of the results. Sensitivity analyses will be conducted to address this limitation.  CPRD is a UK database limiting generalizability to other countries. In addition, it may not be possible to rule out the impact of unmeasured confounding factors on the primary/secondary objectives although we will adjust for all the measured confounding factors through the inverse probability weighting. |
| References 1. Kundur AR, Singh I, Bulmer AC. Bilirubin, platelet activation and heart disease: A missing link to cardiovascular protection in Gilbert’s syndrome? Atherosclerosis. 2015.  2. Wagner K-H, Wallner M, Mölzer C, Gazzin S, Bulmer AC, Tiribelli C, et al. Looking to the horizon: the role of bilirubin in the development and prevention of age-related chronic diseases. Clin Sci. 2015;  3. Park S, Kim DH, Hwang JH, Kim YC, Kim JH, Lim CS, et al. Elevated bilirubin levels are associated with a better renal prognosis and ameliorate kidney fibrosis. PLoS One. 2017;  4. Riphagen IJ, Deetman PE, Bakker SJL, Navis G, Cooper ME, Lewis JB, et al. Bilirubin and Progression of Nephropathy in Type 2 Diabetes: A Post-Hoc Analysis of RENAAL with Independent Replication in IDNT For Peer Review Only Diabetes.  5. Inoguchi T, Sasaki S, Kobayashi K, Takayanagi R, Yamada T. Relationship Between Gilbert Syndrome and Prevalence of Vascular Complications in Patients With Diabetes. JAMA [Internet]. 2007 Sep 26;298(12):1396. Available from: http://jama.jamanetwork.com/article.aspx?doi=10.1001/jama.298.12.1398-b  6. Mashitani T, Hayashino Y, Okamura S, Tsujii S, Ishii H. Correlations between serum bilirubin levels and diabetic nephropathy progression among japanese type 2 diabetic patients: A prospective cohort study (diabetes distress and care registry at tenri [DDCRT 5]). Diabetes Care. 2014;  7. Wagner KH, Shiels RG, Lang CA, Seyed Khoei N, Bulmer AC. Diagnostic criteria and contributors to Gilbert’s syndrome. Critical Reviews in Clinical Laboratory Sciences. 2018.  8. Schoenfeld DA, Schoenfeld DA. Sample-Size Formula for the Proportional-Hazards Regression Model. 2008;39(2):499–503.  9. Karim ME, Gustafson P, Petkau J, Zhao Y, Shirani A, Kingwell E, et al. Practice of Epidemiology Marginal Structural Cox Models for Estimating the Association Between β -Interferon Exposure and Disease Progression in a Multiple Sclerosis Cohort. 2014;180(2):160–71.  10. Ali M, Altman DG. Inverse probability weighting. 2016;189(January):1–2. Available from: http://dx.doi.org/doi:10.1136/bmj.i189 |
| List of Appendices Appendix 1 Inclusion exclusion flow chart for the diabetic cohort (CH1)  Appendix 2 Inclusion exclusion flow chart for the hypertension cohort (CH2)  Appendix 3 List of diabetic medication  Appendix 4 List of Read code for Essential Hypertension  Appendix 5 List of Read code for liver damage  Appendix 6 List of Read code of Anaemia  Appendix 7 List of BNF chapters for the HIV treatment  Appendix 8 List of BNF chapters for the malignancy treatment  Appendix 9 List of UGT1A1 inhibitors (list obtained from WHODrug Standardised Drug Groupings)  Appendix 10 List of covariates |

**Amendment -February 12^th^ 2021
E. Objectives, Specific Aims and Rationale,**

Objective for post-hoc analysis:

- To investigate the diabetic disease progression in relation to bilirubin levels in type2 diabetic patients.

| **Objective for post-hoc analysis**  4) To investigate the diabetic disease progression in relation to bilirubin levels in type2 diabetic patients, in a real-world setting. | **Outcome measure**  - Time to first introduction of additional anti-diabetic medication.  - Time to start of the insulin therapy | **Hypothesis tested**  4) Naturally elevated serum bilirubin level of type2 diabetic patients is associated with slower diabetic disease progression when measured by additional anti-diabetic medication therapy. |
| --- | --- | --- |

Rational:

In the analyses based on the original protocol, we found that high circulating bilirubin is associated with reduced renal diseases risk. We have based this analysis on two patient populations at risk of renal function decline, diabetes and hypertension. We have expected a much higher risk reduction for the diabetic patient cohort as bilirubin is known to increase insulin sensitivity, so the slower loss of glycemic control and bilirubin’s direct protective effect (through anti-oxidative effect) will reduce the kidney function decline. However, we did not find differences in risk reduction for both cohorts on the primary endpoint (eGFR decline, HR 0.88 for diabetic cohort and HR 0.91 for hypertensive cohort), while there was a larger risk reduction on the hypertensive cohort on the secondary endpoint (albuminuria, HR 0.89 for diabetic cohort and 0.82 for hypertensive cohort). This unexpected finding has made us required to directly evaluate bilirubin’s association with the key differences between these two cohorts (one cohort having normal glycemic control while the other has worse glycemic control)

Thus we propose a posthoc analysis of the associations between loss of glycemic control and circulating bilirubin. To reliably quantify the patients’ loss of glycemic control, we have chosen a commonly used endpoint in the clinical trials, “introduction to additional anti-diabetic therapy”, as an endpoint.

## Exposures, Outcomes and Covariates

**Outcome:**

Outcomes will include the following events:

Additional objective: diabetic disease progression

1) Time to first introduction of additional anti-diabetic medication.

- All anti-diabetic medication as listed in Appendix 3

- Insulin
