# Supplementary material for: Bilirubin levels and kidney function decline: An analysis of clinical trial and real world data
Source: PLoS One. 2022 Jun 21;17(6):e0269970. doi: 10.1371/journal.pone.0269970 (PMC9212140; doi:10.1371/journal.pone.0269970)
Supplement: S1 Table — Number of patients with missing covariate records at baseline. † Record not available for CPRD ‡ Record not available for SAVOR. (DOCX) [file pone.0269970.s002.docx]

|  | SAVOR | | CPRD Diabetic | | CPRD Hypertensive | |
| --- | --- | --- | --- | --- | --- | --- |
|  | **Low Bilirubin** | **High Bilirubin** | **Low Bilirubin** | **High Bilirubin** | **Low Bilirubin** | **High Bilirubin** |
| Body Mass Index (kg/m^2) | 10 (0.2) | 5 (0.1) | 3665 (10.6) | 3594 (9.9) | 12882 (31.4) | 13484 (32.8) |
| Diastolic blood pressure (mm Hg) | 1 (0.0) | 2 (0.0) | 336 (1.0) | 300 (0.8) | 87 (0.2) | 78 (0.2) |
| eGFR CKDEPI | 4 (0.1) | 5 (0.1) | 35 (0.1) | 34 (0.1) | 146 (0.4) | 211 (0.5) |
| Fasting Plasma Glucose (mg/dL)^†^ | 162 (3.3) | 146 (2.6) | - | - | - | - |
| Fasting prior to baseline measurement^†^ | 8 (0.2) | 1 (0.0) | - | - | - | - |
| HbA1c (%) | 16 (0.3) | 20 (0.4) | 347 (1.0) | 395 (1.1) | 34308 (83.7) | 35098 (85.5) |
| HDL cholesterol (mmol/L) | - | - | 2421 (7.0) | 2168 (6.0) | 5212 (12.7) | 4180 (10.2) |
| Serum Insulin (pmol/l) ^†^ | 251 (5.1) | 238 (4.2) | - | - | - | - |
| LDL cholesterol (mmol/L)^‡^ | - | - | 6596 (19.0) | 6079 (16.7) | 9540 (23.3) | 8013 (19.5) |
| Protein Dipstick Test^‡^ | - | - | 25206 (72.6) | 25514 (70.1) | 34676 (84.6) | 34490 (84.0) |
| Serum Albumin (g/L) ^‡^ | - | - | 916 (2.6) | 816 (2.2) | 1153 (2.8) | 840 (2.0) |
| Serum Creatinine(µmol/L) | 4 (0.1) | 5 (0.1) | 35 (0.1) | 34 (0.1) | 146 (0.4) | 211 (0.5) |
| Serum Potassium (µmol/L) ^‡^ | - | - | 139 (0.4) | 140 (0.4) | 305 (0.7) | 340 (0.8) |
| Serum Sodium (µmol/L) ^‡^ | - | - | 177 (0.5) | 179 (0.5) | 334 (0.8) | 371 (0.9) |
| Serum Uric Acid (µmol/L) ^‡^ | - | - | 32477 (93.5) | 33985 (93.4) | 38353 (93.6) | 38378 (93.4) |
| Smoking | 0 (0.0) | 0 (0.0) | 1512 (4.4) | 1576 (4.3) | 1942 (4.7) | 2128 (5.2) |
| Systolic blood pressure (mm Hg) | 1 (0.0) | 2 (0.0) | 336 (1.0) | 300 (0.8) | 87 (0.2) | 78 (0.2) |
| Urine Albumin Creatinine ratio (g/mol) | 131 (2.7) | 138 (2.5) | 14658 (42.2) | 15646 (43.0) | 37135 (90.6) | 37669 (91.7) |
