# Supplementary figures and images for: Bilirubin levels and kidney function decline: An analysis of clinical trial and real world data
Source: PLoS One. 2022 Jun 21;17(6):e0269970. doi: 10.1371/journal.pone.0269970 (PMC9212140; doi:10.1371/journal.pone.0269970)

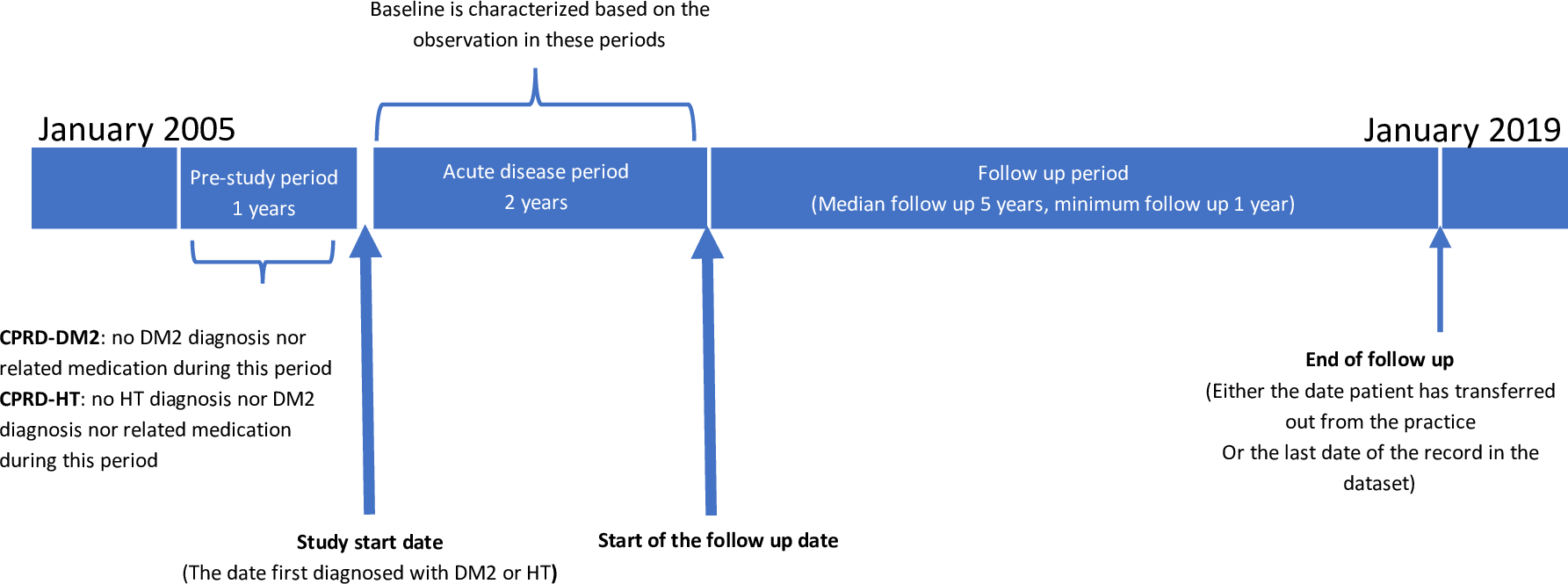

Supplement: S1 Fig — (TIF) [file pone.0269970.s003.tif]

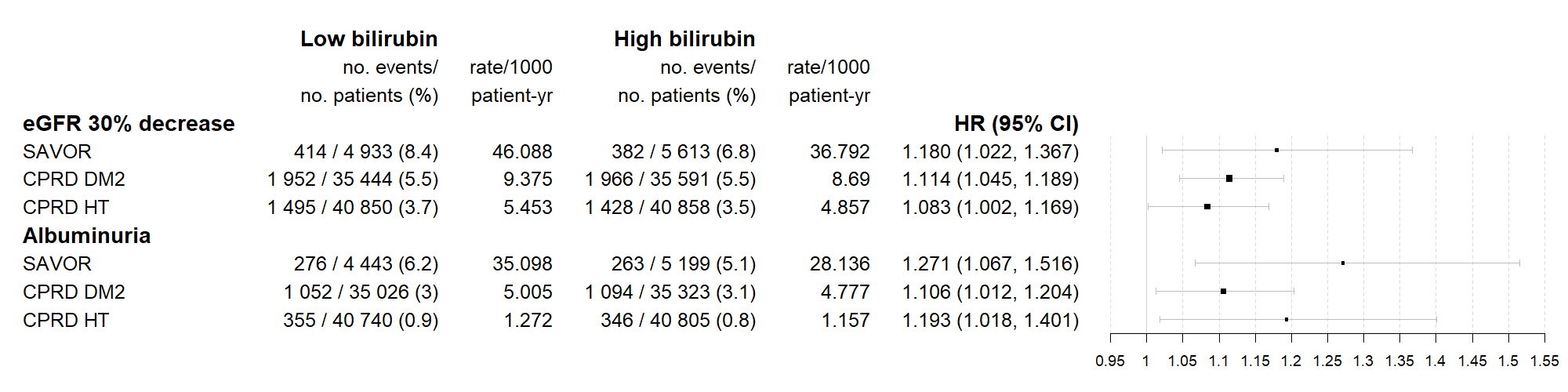

Supplement: S2 Fig — Shown is the primary endpoint of the study -estimated glomerular filtration rate (estimated by the CKD EPI formula) of more than 30% from the baseline- and the secondary endpoint of the study -first observation of the albuminuria (defined by the urine albumin creatinine ratio greater or equal to 30mg/mmol)- according to the study cohorts: SAVOR, a subset of The Saxagliptin Assessment of Vascular Outcomes Recorded in Patients with Diabetes Mellitus-Thrombolysis in Myocardial Infarction 53 trial, CPRD-DM2 (Type 2 diabetic cohort constructed from Clinical Practice Research Datalink), CPRD-HT (Hypertensive cohort constructed from Clinical Practice Research Datalink). High bilirubin denotes the patient subpopulation that has above or equal cohort median of the baseline serum bilirubin concentration (9μmol/L for SAVOR, 10μnil/L for CPRD-DM2 and HT), Low bilirubin denotes the patient subpopulation that has below cohort median of the baseline serum bilirubin concentration, HR hazard ratio adjusted for confounding covariates (age, sex, race if available, baseline body mass index, baseline hemoglobin, baseline alanine transaminase, baseline aspartate transaminase, smoking), CI confidence interval calculated by fitting a normal distribution to the 1000 bootstrap sample of the HR. (TIF) [file pone.0269970.s004.tif]

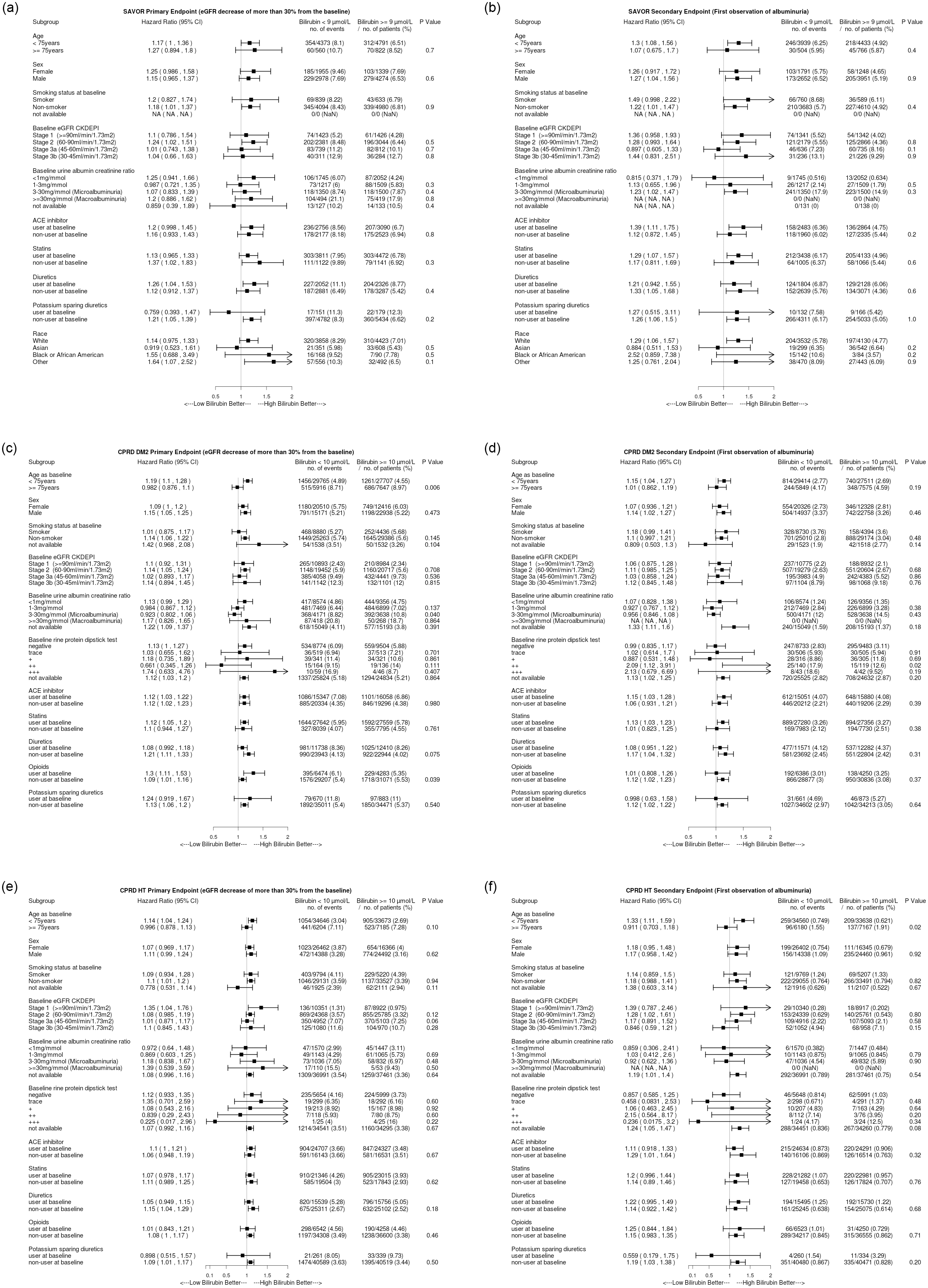

Supplement: S3 Fig — a. Prespecified subgroup analyses of Primary endpoint of SAVOR cohort, Hazard Ratio calculated with inverse probability weighting without multivariable adjustment. b. Prespecified subgroup analyses of Secondary endpoint of SAVOR cohort, Hazard Ratio calculated with inverse probability weighting without multivariable adjustment. c. Prespecified subgroup analyses of Primary endpoint of CPRD-DM2 cohort, Hazard Ratio calculated with inverse probability weighting without multivariable adjustment. d. Prespecified subgroup analyses of Secondary endpoint of CPRD-DM2 cohort, Hazard Ratio calculated with inverse probability weighting without multivariable adjustment. e. Prespecified subgroup analyses of Primary endpoint of CPRD-HT cohort, Hazard Ratio calculated with inverse probability weighting without multivariable adjustment. f. Prespecified subgroup analyses of Secondary endpoint of CPRD-HT cohort, Hazard Ratio calculated with inverse probability weighting without multivariable adjustment. (TIF) [file pone.0269970.s005.tif]
